# Sequence effects and SSD are associated with N2 and P3 signals during stopping in a visual stop signal task

**DOI:** 10.3389/fnhum.2026.1906495

**Published:** 2026-10-12

**Authors:** Knut Andre Skaug, Lars Vidar Magnusson, Rene Jürgen Huster

**Affiliations:** 1 Department of Psychology, University of Oslo, Oslo, Norway; 2 Department of Computer Science and Communication, Østfold University College, Halden, Norway

**Keywords:** EEG, ERP, N2, P3, sequence effects, SSD, stop signal task, successful and unsuccessful stopping

## Abstract

Studies combining the stop signal task (SST) with Electroencephalogram (EEG) have often asked whether the frontocentral N2 and P3 components are influenced by failed versus successful stopping. Estimates of the N2 and P3 commonly reflect averages across experimental trials per individual allowing for assessing mean-level effects. The probability of responding to a stop trial is influenced by task parameters such as the stop signal delay and the asymmetric presentation frequency of Go and Stop trials. Trial-by-trial variation in task parameters like the stop signal delay have been shown to influence brain activity differences between stop outcomes in fMRI data but have not been investigated in EEG data. Changes in the global frequency of Go and Stop trials have been shown to influence behavioral performance and the N2 and P3 components but potential influences of local sequential dependencies that may arise on a trial-by-trial basis have simply been ignored. It is possible that both the SSD and local sequential dependencies arising from past trial histories systematically influence N2 and P3 amplitudes or latencies during stopping. This study used mixed effects modeling to assess associations between SSD, sequential dependencies, and single trial estimates of latency and amplitude of the N2 and P3 components during failed and successful stopping. We specified interactions between both predictor variables and the two stop outcomes. The results showed complex curvilinear associations between the latency of the N2 and P3 components with SSD. Longer SSDs were associated with shorter latencies while only the N2 showed significant interaction with stop outcomes. Sequential dependencies were significantly associated with the N2 and P3 amplitudes. A longer series of go trials was associated with more negative N2 amplitudes and more positive P3 amplitudes. The N2 amplitude was larger after having recently encountered a stop trial, while the P3 amplitude increased at a similar rate during both failed and successful stopping. Together, these results indicate that accounting for the influence of SSD and sequential dependencies on ERP estimates of the N2 and P3 components is of high importance, particularly in studies comparing clinical to healthy control participants.

## Introduction

Our ability to adapt our actions to changing environmental demands is likely to involve higher-order cognitive control functions (Gratton et al., 2018). Response inhibition is a key concept within cognitive control encompassing our ability to suppress unwanted actions (Huster et al., 2013). Many have explored whether deficits in inhibitory control (i.e., response inhibition) may explain impulsive behavior seen in different psychiatric disorders (Bari and Robbins, 2013; Dalley et al., 2011). An underlying question is whether stopping a simple motor response captures a “stopping mechanism” that generalizes to mental processes involved in more cognition-like inhibition (Anderson et al., 2025; Wessel and Anderson, 2024). As inhibitory control in one form or another is shared among many different models of psychiatric disorders (Wessel and Anderson, 2024), a motivating factor behind research probing response inhibition is that it may offer a better understanding of processes associated with distressful symptoms. The Stop signal task (SST) has been a widely used paradigm for studying individual differences in response inhibition (Verbruggen et al., 2019; Verbruggen and Logan, 2008). Participants have to balance two competing goals; (1) responding as fast as they can to go signals, and (2) inhibiting that response when a stop signal occurs. An accumulated response tendency is created by presenting the go signal on every trial while a stop signal follows the go signal only on a minority of trials. Response inhibition is required when a stop stimulus signals the need to inhibit the response tendency during stop trials. To vary the difficulty of stopping, the task dynamically controls the interval between the go and the stop signal. This interval is called the stop-signal delay (SSD). Successful stopping increases the SSD, making it harder on the upcoming stop trial, while unsuccessful stopping decreases the SSD, making it easier to stop. This tracking procedure typically results in an approximately 50 percent (0.5) success rate. Consequently, SSD is the primary task parameter for adjusting the difficulty of stopping in the SST within a given task context. Individual differences in inhibitory capacity are typically evaluated under the assumption that the average SSD approximates the point for which the 0.5 success rate is located.

Because a stop signal instructs participants to refrain from responding, successful stopping is defined by the absence of a response. The independent horse-race model is a stochastic model that offers an estimation of the latency of the stopping process based on a combination of response times to go stimuli, SSDs, and the probability of responding given a stop signal (Logan and Cowan, 1984). In this model stopping fails if the “go-process” finishes before the “stop process” while stopping succeeds if the stop process wins the race (Verbruggen and Logan, 2008). The unobserved stopping latency, called the stop signal response time (SSRT), allows for statistical evaluation of between-group contrasts as well as associations with symptoms scores in clinical studies or brain activity in studies collecting imaging or electrophysiological (EEG) data during the SST.

Numerous factors have been shown to influence behavioral performance in the SST often by influencing one or several parameters necessary for estimating the SSRT or the estimated SSRT itself. Commonly explored factors involve rewarding schemes, foreknowledge of stopping probability and the base rates of stop/go trials. Several studies have found evidence supporting that contextual cues or foreknowledge of the likelihood of a stop signal on the upcoming trial can slow down go-reaction times (Messel et al., 2019; Vink et al., 2015). Foreknowledge produces inconsistent results on SSRTs, which may be explained by experimental designs, with event-related designs producing faster SSRTs and slower go reactions relative to blocked designs where foreknowledge or no-foreknowledge is separated by experimental blocks (Gordi et al., 2019). Studies that vary the proportion (i.e., base rate) of stop/go-trials show that conditions of higher stop trial probability slow down go-reaction times (Doekemeijer et al., 2023; Ramautar et al., 2004), however, only in the study by Doekemeijer et al. (2023) did participants’ SSRTs speed up. One study using computational modeling further indicates that go-reactions are slowed down with increased stop trial probability as well as increased SSDs, and that each predictor has an independent influence on go reaction time variability (Ma and Yu, 2015). Yet another group using computational modeling showed that while increased probability of having to stop slows down Go RT, a longer motor preparation time may influence this relationship by decreasing Go RT – suggesting that the two possibly anticipatory effects may interact (Wang et al., 2018). Interestingly, both computational modeling studies further indicate that not everyone is equally influenced by factors observed to modulate go-reaction times (Ma and Yu, 2015; Wang et al., 2018). Some individuals show a slowing of go-reaction times with increasing stop signal probability (Ma and Yu, 2015; Wang et al., 2018) while others are more influenced by SSDs (Ma and Yu, 2015). Perhaps a stop stimulus sometimes reflects a confirmation of expectations while at other times it introduces a conflict between goals (i.e., going and stopping). Studies assessing the effects of motivating either going over stopping or vice versa have further demonstrated that performance in the SST is partly shaped by contextual cues. Although inter-individual differences were not tested specifically, mean-level effects of reward schemes indicate that motivating going over stopping increased the speed of go reaction times (Giuffrida et al., 2023), and that this increased speed was also present for unsuccessful stop trials suggesting that participants overall went faster (Greenhouse and Wessel, 2013). Interestingly, Giuffrida et al. (2023) showed that SSDs became slower when stopping was rewarded and that SSDs, but not SSRTs, were highly correlated with go reaction times. Apparent from previous studies using variants of the SST is that numerous contextual and motivational factors can introduce variation in behavioral performance which may influence estimates of SSRTs. In addition to changes in the underlying terms of the SSRT estimation, a number of these studies highlight the potential role statistical regularities, or accumulated dependencies, may have on response tendencies in the SST. It is largely unknown to what extent individuals revise expectations about upcoming events in the SST given prior sequences of events, and whether sequential dependencies impact subsequent responses.

An often-ignored aspect in behavioral or combined EEG-behavioral analyses of the SST is that repeatedly responding to stimuli organized in a trial structure may produce sequential dependencies that carry influence on future choices. Sequential effects have been observed across different response time paradigms, focusing on the effect the previous trial type has on the current response (Voormann and Miller, 2024). For example, being faster when dimensions of the previous trial repeat, or slower when they alternate. Key to understanding sequence effects is that they may arise in what is presumed to be a purely random sequence (Meyniel et al., 2016). Thus, even though the SST typically ensures that trial orders of Go and Stop trials are randomly distributed, it can only last for a set duration, resulting in sequences that repeat trial types when local histories are considered. The easiest way to index local trial histories would be to count the number of go trials between any two stop trials. By limiting each sequence to include four to five trial-long histories, certain trial sequences occur repeatedly throughout the task. Sequential dependencies arise if an increasing number of go trials are systematically associated with brain activity or behavioral outcomes. Put simply, if the time since the last stop trial encountered matters for the current stop trial. Whether sequence effects from local histories of trial types interfere with the bias towards going in the SST is largely unknown, at least when considering longer sequences of trials. In other cognitive control tasks, like the Flanker task (i.e., interference) or the Stroop task (i.e., task-switching), sequence-congruency effects are observed as less slowing (i.e., smaller congruency effect) on the current incongruent trial if the previous trial was incongruent (as opposed to congruent) (Egner, 2007). This suggests that the level of conflict on incongruent trials may be influenced by preceding trial sequences. Most studies that have investigated sequential effects in the SST limited the search of prior influence to include only the previous trial’s influence on the current trial (Bissett and Logan, 2011, 2012a, 2012b; Rieger and Gauggel, 1999). Response slowing was observed across studies when highly local sequences were considered leaving the possibility that also longer sequential dependencies may influence the probability of successful stopping. Beyond purely behavioral investigations, these findings open the possibility that also common EEG signatures of stopping are sensitive to sequential effects in the SST.

Commonly investigated EEG components associated with stopping in the SST are modulated by similar contextual factors to those also modulating behavioral performance (Greenhouse and Wessel, 2013; Huster et al., 2013; Ramautar et al., 2004; Waller et al., 2021). An extension of these findings is that individual differences due to contextual and motivational variables may influence brain processes giving rise to the ERP when averages are formed across trials. Of these commonly investigated components, the N2 and P3 components at fronto-central electrodes are most often estimated to assess inhibitory differences using the SST (Greenhouse and Wessel, 2013; Huster et al., 2020; Senderecka, 2016; Bekker et al., 2005). N2 denotes a negative deflection around ~200 ms while the P3 denotes a positive deflection around ~300 ms. Both ERPs show larger phase consistency across lower frequency ranges, like the delta (P3) and theta (N2) ranges (Huster et al., 2017). Unfortunately, large methodological variation between studies in how component scores were estimated and how they were statistically evaluated leave the expected pattern of effects hard to determine before conducting a SST study. No EEG study has, for example, investigated whether either component scales with the primary experimental variable (i.e., SSD), designed to increase or decrease the probability of successful stopping in a given stop trial. One study that specifically asked whether fMRI activity differences between fast and slow participants was confounded by absolute SSD differences, showed that SSD was a confounding factor on the group contrast (D'Alberto et al., 2018). Thus, group differences in average brain activity associated with stopping performance could partly be explained by absolute differences in the SSDs experienced by the participants. It is therefore important to evaluate whether the N2 and P3 covary with the primary experimental variable SSD designed to make stopping easier or harder. Furthermore, disentangling the effects related to SSD and subsequent stopping is a large methodological challenge when using fMRI due to its limited temporal resolution (Lin et al., 2026). The increased temporal resolution of EEG provides a better opportunity to investigate whether specific brain processes vary with SSD.

Sequential effects on P3 amplitudes suggest that this signal is highly sensitive to serial dependence developed over a local history of trials with increasing amplitudes when the current trial violated past stimulus sequences (Squires et al., 1976). The P3 is sensitive to both first- and second-order stimulus repetition and alternations, further indicating its role in keeping track of regularities across time (Kolossa et al., 2013). Similarly, a study using the go/no-go task showed that delta band activity scaled with the number of go trials preceding a no-go trial while theta band activity did not (Harper et al., 2016). Although these contextual differences are global, meaning that local sequential effects cannot be easily dissociated, the results suggest that the P3 component is at least sensitive to dependencies developed from the stimulus context. Assessing the partial influence of sequential dependencies on P3 effects in cognitive control tasks is therefore important to contrast against variation possibly driven by conflict. Mapping this potential confounding relationship becomes important particularly in cases where a group contrast is of interest. Besides amplitudes, these sequential dependencies are also found to covary with N2 and P3 latency estimates with complex relationships (Thomas et al., 2009), indicating the need for careful analyses when potential group estimates may be confounded by trial histories (Groenwold et al., 2013).

Both sequential dependencies and SSD may have varying effects on individuals that need to be accounted for when estimating group-level effects. Linear mixed effect models accommodate dependencies across observations, categorical and continuous predictor variables, differences in the effects of task parameters between individuals, and yield robust estimates of between-individual effects in the presence of missing data (Frömer et al., 2018; Barr, 2013; Matuschek et al., 2017). However, linear-mixed effect modeling does not solve the issue of noisy single trial estimates of ERP components. Latency minimization by means of template matching is a data-driven alternative to permutation testing that may increase the chance of detecting the peak onset of a pre-determined ERP component. Trial-by-trial latency variation (i.e., latency jitter) in time-locked brain activity may bias ERP estimates (i.e., averaged waveform across trials) of both amplitude and latency between conditions (Ouyang et al., 2016). Accounting for trial-wise latency variation may reduce between group effects on amplitude responses (Kovarski et al., 2019; Li et al., 2024), help separate simultaneously active processes time-locked to stimuli in cognitive control experiments (Mückschel et al., 2017a, 2017b), and may thus improve the estimation of early and late ERP components, such as the N2 and P3 (Ouyang et al., 2013). Minimizing latency variation can make it easier to estimate both the amplitude and latency of the N2 or P3 embedded in activity not phase-locked to the stimulus. This is particularly useful when modeling associations between task parameters and N2/P3 observations at single trial. We developed a simple method for minimizing latency differences at single trial within individuals to make it easier to estimate when the N2 and P3 components peaked following stop signals. This approach facilitates the investigation into how continuous task parameters in a standard visual SST are associated with the N2 and P3 components time-locked to the stop signal.

In this study, we assessed whether SSD and sequential dependencies are associated with common EEG signatures of stopping. We model single trial estimates of N2 and P3 amplitude and latency using linear mixed effects methods. Specifically, this study evaluates whether SSD is significantly associated with any of the EEG outcome variables. Given the influence of SSD on the probability of successful stopping, EEG components sensitive to differences in task difficulty should covary with SSD. We include trial-sequence dependencies in the model to assess potential relationships with EEG components. Sequence effects are defined up to four trials back extending previous behavioral investigations. Our analysis also asks whether the possible association between SSD or sequence with any of the EEG outcome variables are different between successful and unsuccessful stop trials. By modeling single trials using mixed effects models this analysis allows for statistically controlling the influence of each variable on the other variables. Our modeling yielded estimates of the variation underlying differences between successful and unsuccessful stopping while controlling for both SSD and sequence effects. This represents an important step towards understanding how both task parameters drive N2 and P3 component variation during stopping in the SST.

## Method

### Participants

A total of 43 individuals (age; M = 25.95, SD = 3.04, sex; male = 15, female = 28) participated in a study collecting SST data in combination with EEG. All gave informed consent to allow the use of their data for research objectives approved by the regional ethical committee in Norway. The Norwegian Centre for Research Data (NSD) approved the data management plan.

### Stop signal task

The visual stop signal task comprised 5 blocks presenting a total of ~330 go trials and ~110 stop trials. All stimuli were presented at the center of the screen. The size of the stimuli was 10.5 cm x 6 cm and participants were seated ~60 cm away from the screen. A go trial comprised a black right- or left-pointing arrow presented for 100 ms. A stop trial presented the same arrow in either blue or orange color for 100 ms. The direction and color were counterbalanced across blocks to minimize response tendencies associated with either hand or color. A central fixation cross was presented prior to any go arrow. For each block 24.4% of the trials were stop trials ensuring a response bias towards go trials. A pseudorandom number generator produced the sequences of trial types for each block with a limitation that the first trial in each block was always a go trial and the first ten trials of the first block were always go trials. The order of which trials appear is randomized within the boundaries of these constraints. Participants were not informed about the number of trials in each block before starting the experiment, reducing the possibility of calculating the probability of observing a specific trial given the history of past trial types. An adaptive staircase increased or decreased the SSD in steps of 50 ms with a lower bound of 100 ms and upper bound of 600 ms. The staircase stepped up or down regardless of whether the trial required a left- or right-hand response. An error in the experimental script resulted in SSDs taking on the value of 120 ms for most trials that should have been 100 ms. Three trials across all individuals had an SSD of 100 ms. Before the task began, participants read instructions explaining how to perform the SST and emphasizing response speed and de-emphasizing slowing for the verbal instructions given. All performed a short practice phase before starting the experiment. The experiment was coded using functions from PsychToolbox-3 (version 3.06.16) in MATLAB R2018b (The MathWorks, Inc., Massachusetts, United States) and presented on an Eizo FlexScan S2411W monitor (60 Hz refresh rate) connected to a Dell Precision T5500 computer.

### Behavioral descriptives

We calculated success rate and average SSD for each participant and the average for both measures across participants. The average success rate across individuals was 52.56, 95% CI = [50.35, 54.77]. Three participants had a success rate above 65% and one participant below 35%. The three participants with >65% success rate experienced more than 85% stop trials with SSDs above 450 ms with an average SSD of 551.21 ms. The average SSD for the participant with the lowest success rate was 155 ms. The remaining 39 participants had an average SSD of 357.14 ms. More extreme SSDs together with higher/lower success rates indicate that these participants performed the task differently from most participants. We also calculated average reaction times for Go trials and unsuccessful stop trials separately for each individual and averaged across all mean RT values. Mean Go RT excluding the outliers was 550.16 ms (SD = 107.61 ms), while mean Go RT for the three outliers with the highest accuracy was 752.12 ms (SD = 145.97 ms). Mean Go RT during unsuccessful stop trials excluding the outliers was 503 ms (SD = 73.15 ms), while mean Go RT during unsuccessful stop trials for the three outliers was 674.18 ms (SD = 95.21 ms). Mean Go RT for the participant with the lowest accuracy was 381.11 ms (SD = 72.77 ms), and the mean Go RT during unsuccessful stop trials was 337.45 ms (SD = 47.58 ms). The identified potential outlier participants were included in the analysis and were only considered outliers if the models produced large deviating estimates for these participants. The average SSD within and across participants alongside the mean success rate and the reaction times suggest that the staircase procedure worked as intended.

### EEG recording settings

A BrainAmp system (Brain products GMBH) was used to record EEG with 31 passive scalp channels (Ag/AgCI) positioned according to the international 10–20 system. All recordings were done in an electrically shielded room. The sampling rate was 5,000 Hz, Fpz was used as online reference, and AFz was used as ground electrode. Before recording any data, all channel impedance estimates were checked to be below 5 kΩ.

### EEG signal processing

#### Preprocessing

EEGLAB v2022.0 (Delorme and Makeig, 2004) was used during fully automated preprocessing in MATLAB (The MathWorks Inc., 2024). Continuous EEG data was resampled to 512 Hz, high-pass filtered at 0.75 Hz, and low-pass filtered at 40 Hz. Independent component analysis (i.e., extended Infomax ICA) decomposed the continuous data into components. IClabel was used to identify components with >.75% likelihood of containing muscular and >0.35% likelihood of containing ocular artifacts. Identified components were projected out from the data. An in-house script was used to identify bad channels and EEGLAB was used to spherically interpolate the identified channels. The script involves evaluating whether the combination of the power spectral density and the variance for any channel deviate from the general variance or power spectral density across all channels. All active scalp channels were next re-referenced to the average of the two mastoid channels. The last preprocessing step involved again identifying and interpolating any remaining bad channels. No trials were excluded during preprocessing, and the data was kept continuous for the next step which was to minimize inter-trial latency variation.

#### Template matching to minimize inter-trial latency variation

The idea behind our template matching procedure was to minimize latency jitter and thereby increase the chance of capturing the N2 and P3 component peak amplitude and latency at the single trial level. A complete description of the steps in the procedure is found in the Supplementary material. In short, by aligning trials through cross-correlation we could first identify the time point for each component, then use this time point and the relative shift of each single trial to locate the amplitude in unshifted single trial. The whole template matching is done on a copy of the dataset, amplitude and latency were extracted from the unshifted version of the dataset. Importantly, for both datasets, the continuous data was low-pass filtered at 12 Hz, and high-pass filtered at 1.5 Hz before segmenting the data according to stop stimulus onsets. Any amplitude value reflects signal characteristics within this frequency range. Previous research shows that most of time-locked variation of the N2 and P3 components reside well within this upper and lower frequency range (Huster et al., 2017). Windows used for segmenting ranged from −1 s to 1 s. The template matching procedure was carried out on these segments by minimizing the latency jitter between all single trials. The procedure first builds an average template from minimizing latency jitter between single trials, before estimating the cross-correlation between each single trial and the new template. The average of the shifted version of the data is compared against the initial average to estimate the mean-square error on the first iteration. The next iterations compare the average of the shifted data against the previous template. Once the procedure no longer results in any significant shifts, the difference between the previous and current iteration will be negligible and the procedure stops. An additional stopping criterion was that no shifts can be below 30% of the SSD for each trial or above 400 ms relative to the initial stimulus onset time. Because no true reference signal exists, we evaluated the procedure by relying on visual inspection of single trials which suggested that the method does reduce temporal variability (see example figure in Supplementary material) but is by no means a perfect method. We argue that it is more likely that the probable N2 and P3 components are identified at single trial level when using this procedure especially for cases when there is large inter-trial variation in when the N2 and P3 occurs. The procedure was developed specifically for the signal of interest in this study and would most likely have to be adapted to other signals of interest in other studies. We conducted a simulation analysis to test the robustness of the template matching procedure (see Supplementary material section 4).

#### Peak detection for identifying the N2 and P3 components

Latency minimization is the first step. Peak identification is the second. We believe that the combined procedure increased the probability of identifying an accurate representation of the evoked signal at single trials. The MATLAB function “findpeaks” was used to identify peaks above a set threshold of 1uV (see Supplementary material for complete description of peak detection). First, for each subject, the N2 and P3 were identified from the average ERP. This allowed an informed time range for where to expect the N2 and P3 to occur at single trial per subject. Z-scored, time-corrected, single trials were used to identify the N2 and P3 peak. Peaks falling inside an interval of 100 ms before/after the N2 latency window of the average ERP were considered N2 peaks. The minimum inside this interval was selected. Once the peak time points were identified, the amplitude and latency of the corresponding unshifted data were extracted ensuring uncorrected variability in both peak onsets and amplitudes. The same peak identification procedure was done for the P3 component albeit instead of minimum, the maximum was used.

### Data analysis

#### Outlier exclusion at single trial level

Criteria for excluding single trial observations were: (1) Any positive N2 amplitudes or negative P3 amplitudes. (2) Any N2 latencies <80 ms or any P3 latencies <120 ms. (3) Any P3 latencies <N2 latencies. If any observation fitted any of these conditions, it was excluded from the analysis. A total of 38 trials were excluded across all subjects. Three trials had an SSD below 120 ms and were excluded. Stop trials where responses preceded the stop signal were excluded (240 trials). A total of 80 trials had missing values in the N2 dataset, while 49 trials had missing values in the P3 dataset. Missing values occurred when no peak was identified. These were excluded during modeling leaving 4,369 trials in the N2 dataset and 4,400 in the P3 dataset.

#### Predictor variables

The continuous predictor variable SSD was first mean-centered across observations and re-scaled to values between −5 and 5. Re-scaling was done to make a one-unit change more interpretable and to decrease the numerical distance between predictor variables. A one-unit change corresponds to 50 ms in the original SSD variable. The predictor variable Sequence was treated as a continuous variable and mean centered across observations. Sum-coded dummy variables were used for the categorical predictor variable Stop outcome, where unsuccessful stopping was assigned a value of 1, and successful stopping was assigned a value of −1. Sequences were defined at the level of trial type (Go vs. Stop trial). We counted the number of go trials between two stop trials disregarding experimental blocks. When two or more stop trials followed in succession, stop trials not preceded by any go trials were counted as the same instance. When four or more go trials preceded a stop trial this was counted as the same instance. All other sequences were counted as unique instances. For example, one sequence could be Stop – Go – Go – Go – Stop – Stop. The first stop trial following go trials would be preceded by three go trials while the last stop trial would be preceded by a stop trial. If three stop trials followed in succession, the latter two would be counted as the same instance. We counted five different instances; when two or more stop trials followed in succession, when one go-trial preceded a stop trial, when two go trials preceded a stop trial, when three go trials preceded a stop trial, and finally when four or more go trials preceded a stop trial.

#### Dependent variables

The latencies and amplitudes of the N2 and P3 components at channel Cz were treated as continuous dependent variables in each separate analysis. We selected Cz to be consistent with past studies investigating the fronto-centrally distributed N2 and/or P3 signals in relation to performance in the SST (Lansbergen et al., 2007; Kok et al., 2004; Greenhouse and Wessel, 2013; Thunberg et al., 2020; Huster et al., 2020; Dimoska and Johnstone, 2008). We visually checked the distributions of each dependent variable and fitted probability density functions to each. Only N2 amplitudes suggested a deviation from normality, and we found that a square root transformation made the distribution normal (see Supplementary material for figures visualizing the distributions of the dependent variables).

#### Linear mixed effect modeling

Statistical modeling was carried out in Rstudio (2024.04.2) (Posit team version, 2024). The package “lme4” was used to fit linear mixed effects models (Bates et al., 2015). To estimate *p*-values using the Satterthwaite method and produce a summary of model coefficients we used the package “lmerTest” (Kuznetsova et al., 2017). The package “emmeans” was used for post-hoc testing of significant interactions (Lenth and Piaskowski, 2025). To visualize models and for other handy functions to work with models, the packages “ggplot2” (Wickham, 2016), “sjPlot” (Lüdecke, 2025a) and “sjstats” (Lüdecke, 2025b) were used. The package “ggeffects” was used to extract marginal effects (Lüdecke, 2018). The package “effectsize” was used to estimate marginal and conditional R^2^ (Ben-Shachar et al., 2020). All models were fitted using the Maximum likelihood estimator. For our model building procedure, we used likelihood tests to evaluate which random effect structure provided the best model fit to our data (Meteyard and Davies, 2020). This was carried out in a stepwise fashion to evaluate different complexity levels of the random effects (see table of model comparison results in Supplementary material). The fixed effect structure was kept constant for this initial step. After selecting the model providing the best model fit, we visually inspected residuals for deviations from assumptions underlying linear mixed effect modeling. We visualized the predicted slopes for each predictor variable against the scores on the dependent variable. In some analyses, this visualization indicated that a nonlinear term for either both or one of the continuous predictor variables might have provided a better model fit to our data. We assess whether including natural cubic splines with 2 degrees of freedom provided a better fit by comparing against a model with linear terms using likelihood ratio tests. We next evaluated whether retaining the interaction terms provided a better model fit using likelihood ratio tests. Having evaluated whether to include splines and to retain interaction terms, we again evaluated which random slope structure provided a better model fit using likelihood ratio tests. The final model was subsequently checked for violation of assumptions by visual inspection of the residuals. We visualized the predicted slopes of the two continuous predictor variables as a function of stop outcome. ANOVA type III was used to evaluate overall effects of predictor variables on the dependent variables, reported in tables below. Model coefficients are reported in separate tables below. Significant interactions were followed up by testing mean-differences across levels of SSD or by a simple slope analysis, using Tukey or Bonferroni corrected *p*-values (Bonferroni are used when more than three means are compared). We approximated marginal and conditional R^2^, pseudo-R^2^ values for each model term, as well as standardized beta coefficients from the final model. Pseudo-R^2^ values and standardized beta coefficients are reported in tables in the Supplementary material. We conducted a sensitivity analysis to evaluate the robustness of the conclusions made from the final model specifications (see Supplementary material).

## Results

### N2 latency

The final model evaluated included a natural cubic spline term for the interaction between SSD and stop outcome and the main effect of SSD, a linear term for the main effect of Sequence and a main effect of stop outcome. The random effect structure included a random intercept for participants, and random slopes for SSD and stop outcome. See Supplementary material for the results from likelihood ratio tests, and a complete description of the steps (see Tables 1, 2).

**Table 1:** Type III ANOVA results (Satterthwaite) for N2 latency.

Effect	F	df num	df den	*p*
SSD	42.75	2	54.6	<0.001***
Stop outcome	30.03	1	167.5	<0.001***
Sequence	0.23	1	4302.5	0.63
SSD*Stop outcome	4.88	2	209.4	0.009**

Significant codes: *** 0.001, ** 0.01, * 0.05.

**Table 2:** Model coefficients for N2 latency.

Effect	Estimate	*SE*	*df*	*t*	*p*
Fixed effects					
Intercept	264.01	4.54	53.66	58.14	<0.001
SSD spline 1	−65.70	7.11	65.35	−9.25	<0.001
SSD spline 2	−14.96	3.71	33.71	−4.04	<0.001
Stop outcome	12.66	2.31	167.53	5.48	<0.001
Sequence	0.18	0.37	4302.51	0.48	0.630
SSD spline 1 * Stop outcome	−3.90	4.79	212.87	−0.82	0.416
SSD spline 1 * Stop outcome	6.17	2.24	175.09	2.76	0.006

Random effects	Variance	SD	Correlation	
Intercept	299.24	17.23		
SSD	2.77	1.66	−0.32	
Stop outcome	9.87	3.14	−0.34	0.53
Residual	1396.65	37.37		

Number of trials = 4,369, number of participants = 43. Unsuccessful stopping = 1, Successful stopping = −1. Satterthwaite’s method used to estimate t-tests.

The estimated marginal R2 was ~10% and the conditional R2 was ~27%, suggesting that most of the variability accounted for by our model was due to individual variability in N2 latency. A type III ANOVA with Satterthwaite estimation of degrees of freedom indicates that both stop outcome (*F*(1,167.1) = 30.02, *p* < 0.001) and SSD (*F*(2,54.6) = 42.75, *p* < 0.001) were significantly associated with N2 latency whereas Sequence was not (*F*(1,4302.5) = 0.23, *p* = 0.630). The interaction between SSD and stop outcome was also significant (*F*(2, 209.4) = 4.87, *p* = 0.009). Follow-up analysis of the interaction by testing mean-differences for each unit (i.e., 11) of the SSD (Bonferroni corrected) indicated that larger differences were found near lower and upper SSD boundaries, with higher SSDs producing larger mean-differences, all *p* < 0.001. Across both stop conditions, lower SSDs were associated with higher N2 latency, while the difference between stop conditions became larger at higher SSDs. After having controlled for the effects of SSD and sequence, unsuccessful stopping lagged successful stopping with approximately 25 ms. The table showing the estimated semi-partial R2 values (see Supplementary material) further indicates that SSD was more strongly associated with N2 latency relative to stop outcome. Although there was a significant interaction between SSD and stop outcome on N2 latency, the mean-differences suggested that the slopes are similar across SSD levels with a slight increased difference near the ends (see Figure 1).

**Figure 1:**
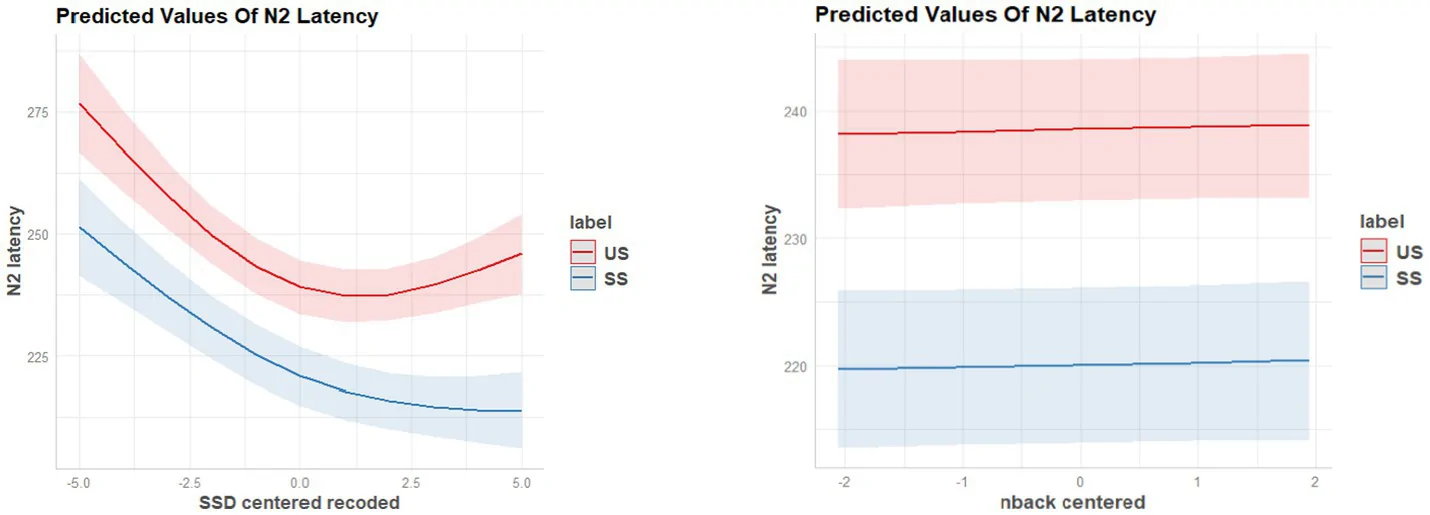
Predicted slopes for SSD and sequence as a function of SS or US. N2 latencies decrease with increasing SSD values and the difference between stop outcomes is larger near the edges. Sequence was not significantly associated with N2 latencies. Unsuccessful stopping lagged successful stopping.

### N2 amplitude

Following model building, the final model included a natural cubic spline term for the main effect of SSD, a linear term for the main effect of Sequence and the interaction between Sequence and stop outcome, and a main effect of stop outcome. The random effect structure included a random intercept for participants, and random slopes for SSD and stop outcome. See Supplementary material for model comparisons and coefficients (see Tables 3, 4).

**Table 3:** Type III ANOVA results (Satterthwaite) for N2 amplitude.

Effect	F	df num	df den	*p*
SSD	38.97	2	80.9	<0.001***
Stop outcome	60.07	1	42.1	<0.001***
Sequence	34.38	1	4294.9	<0.001***
Sequence*Stop outcome	9.23	1	4286.2	0.009**

Significant codes: *** 0.001, ** 0.01, * 0.05.

**Table 4:** Model coefficients for N2 amplitude.

Effect	Estimate	*SE*	*df*	*t*	*p*
Fixed effects					
Intercept	−3.906	0.1501	56.89	−26.017	<0.001
ns (SSD, df = 2)1	−1.794	0.2373	68.12	−7.560	<0.001
ns (SSD, df = 2)2	−0.101	0.1401	45.8	−0.722	0.47
Sequence	−0.054	0.0091	4,295	−5.864	<0.001
Stop outcome	−0.259	0.0335	42.13	−7.750	<0.001
Sequence:Stop outcome	0.028	0.0092	4,286	3.039	0.002

Random effects	Variance	SD	Correlation	
Intercept	0.474	0.689		
SSD	0.008	0.090	0.10	
Stop outcome	0.038	0.195	0.66	−0.06
Residual	0.863	0.929		

Number of trials = 4,369, number of participants = 43. Unsuccessful stopping = 1, Successful stopping = −1. Satterthwaite’s method used to estimate t-tests.

The estimated marginal R^2^ was ~10% and the conditional R^2^ was ~45%, suggesting that a large proportion of variability in N2 amplitudes was captured well by our model and that most of the variability resides within individuals. A type III ANOVA with Satterthwaite estimation of degrees of freedom indicated that SSD (*F*(2,80.9) = 38.97, *p* < 0.001), stop outcome (*F*(1, 42.1) = 60.07, *p* < 0.001), Sequence (*F*(1, 4294.9) = 34.38, *p* < 0.001) as well as the interaction between Sequence and stop outcome (*F*(1, 4286.2) = 9.23, *p* = 0.002), were all significant predictors of N2 amplitudes. A simple-slope (Tukey corrected) analysis of the interaction indicated a significant difference in slopes between unsuccessful and successful stopping, with steeper drop in N2 amplitudes with increasing Sequence for successful stopping, *t*(4286) = 3.039, 95% CI = [0.02, 0.09], *p* = 0.002. Increasing the number of go trials prior to a stop trial is associated with smaller N2 amplitudes, and this decrease in amplitude is steeper for successful stop trials. Approximated semi-partial R^2^ (see Supplementary material) further indicates that the order of effects was such that stop outcome had a higher association with N2 amplitudes whereas the association between N2 amplitudes and SSD was slightly smaller. The lowest order of semi-partial R^2^ was seen for the interaction between Sequence and stop outcome. Stop outcome and Sequence showed a small but significant interaction effect on N2 amplitudes. A simple slope analysis indicated that N2 amplitudes decreased with increasing sequence length. The rate of change was significantly different between successful and unsuccessful stop trials, with successful stop trials showing a larger decrease (see Figure 2).

**Figure 2:**
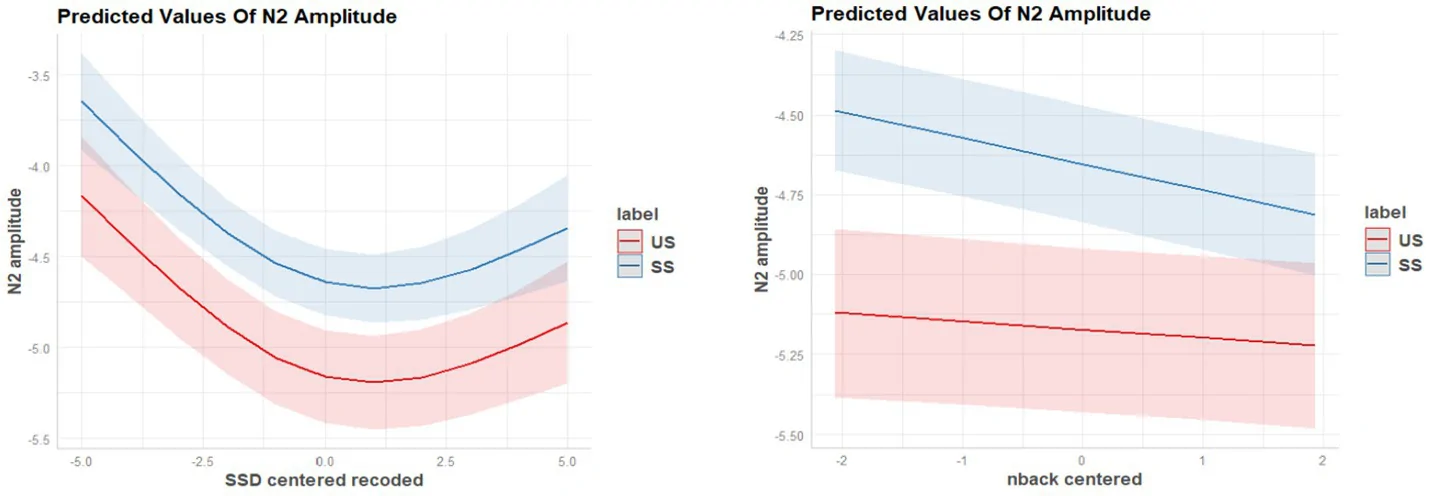
Predicted slopes for SSD and sequence as a function of SS or US. SSD has a curvilinear relationship with N2 amplitudes where the smallest N2 amplitudes coincides with roughly the average SSD. N2 amplitudes decrease with increasing Sequence, and this decrease was larger for successful stop trials relative to unsuccessful stop trials.

### P3 latency

Following model building, the final model included a natural cubic spline term for the main effect of SSD, a linear term for the main effect of Sequence and a main effect of stop outcome. The random effect structure included a random intercept for participants, and random slopes for stop outcome. See Supplementary material for model comparison and coefficients (see Tables 5, 6).

**Table 5:** Type III ANOVA results (Satterthwaite) for P3 latency.

Effect	F	df num	df den	*p*
SSD	43.68	2	3998.9	<0.001***
Stop outcome	119.77	1	47.6	<0.001***
Sequence	2.23	1	4359.1	0.135

Significant codes: *** 0.001, ** 0.01, * 0.05.

**Table 6:** Model coefficients for P3 latency.

Effect	Estimate	*SE*	*df*	*t*	*p*
Fixed effects					
Intercept	397.409	4.9873	109.67	79.684	<0.001
ns (SSD, df = 2)1	−60.400	6.8786	4165.55	−8.781	<0.001
ns (SSD, df = 2)2	−21.221	3.4810	3680.79	−6.096	<0.001
Sequence	−0.629	0.4209	4359.09	−1.493	0.135
Stop outcome	10.579	0.9666	47.62	−10.944	<0.001

Random effects	Variance	SD	Correlation
Intercept	634.52	25.190	
Stop outcome	19.22	4.385	−0.42
Residual	1869.46	43.237	

Number of trials = 4,400, number of participants = 43. Unsuccessful stopping = 1, Successful stopping = −1. Satterthwaite’s method used to estimate t-tests.

The estimated marginal and conditional R2 was ~7% and 31%, indicating that a large proportion of variance in P3 latency in our model resides within individuals. A type III ANOVA indicated that SSD was significantly associated with P3 latency, *F*(2, 3998.9) = 43.68, *p* < 0.001. There was a significant difference in P3 latencies between stop outcomes, *F*(1, 47.6) = 119.77, *p* < 0.001 with faster P3 latency during successful stopping. Sequence was not significantly associated with P3 latency, *F*(1, 4359.1) = 2.23, *p* = 0.135. The estimated semi-partial R2 (see Supplementary material) further indicates that stop outcome was a slightly more important predictor of P3 latency relative to SSD. P3 latency shows a curvilinear relationship with SSD, decreasing faster at lower ends of the SSD with an increase towards the higher SSDs. Unsuccessful stop trials were lagging successful stop trials by an estimated 21 ms difference, after controlling for SSD and Sequence (see Figure 3).

**Figure 3:**
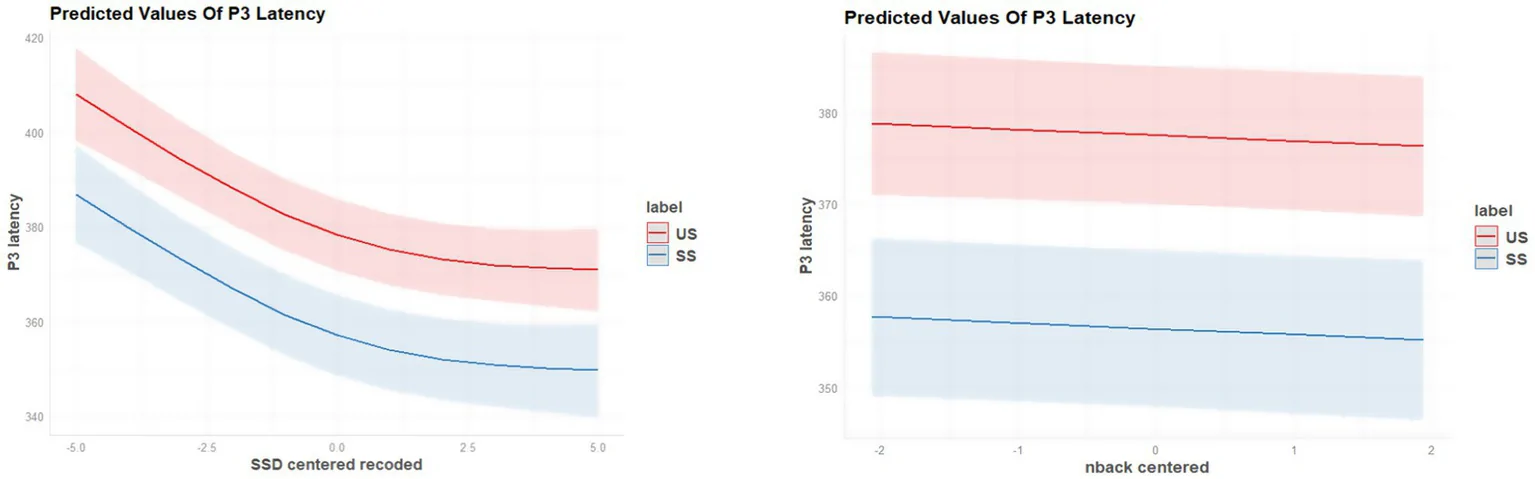
Predicted slopes for SSD and sequence as a function of SS or US.

### P3 amplitude

Following model building, the final model comprised a main effect of SSD, a main effect of stop outcome, a main effect of Sequence and an interaction between Sequence and stop outcome. The random effect structure included a random intercept for participant, and random slopes for SSD, Sequence and stop outcome. See Supplementary material for model coefficients, results from likelihood ratio tests, and a complete description of the steps (see Tables 7, 8).

**Table 7:** Type III ANOVA results (Satterthwaite) for P3 amplitude.

Effect	F	df num	df den	*p*
SSD	0.07	1	43.58	0.791
Stop outcome	3.83	1	41.91	0.057
Sequence	48.33	1	44.17	<0.001***
SSD * Stop outcome	6.30	1	376.08	0.013*

Significant codes: *** 0.001, ** 0.01, * 0.05.

**Table 8:** Model coefficients for P3 amplitude.

Effect	Estimate	*SE*	*df*	*t*	*p*
Fixed effects					
Intercept	25.29	0.89	42.49	28.209	<0.001
SSD	0.036	0.136	43.58	0.267	0.791
Sequence	0.984	0.142	44.17	6.952	<0.001
Stop outcome	0.537	0.274	41.91	1.957	0.057
SSD*Stop outcome	−0.166	0.066	376.08	−2.509	0.013

Random effects	Variance	SD	Correlation		
Intercept	33.13	5.76			
SSD	0.49	0.70	0.30		
Sequence	0.60	0.78	0.30	0.18	
Stop outcome	2.48	1.57	0.56	0.16	0.02
Residual	62.46	7.90			

Number of trials = 4,400, number of participants = 43. Unsuccessful stopping = 1, Successful stopping = −1. Satterthwaite’s method used to estimate t-tests.

The marginal and conditional estimated R^2^ was 3% and 41%, indicating that our fixed effects predictor variables accounted for a very small proportion of the variability in P3 amplitudes relative to the within-individual proportion. After visualizing the predicted slopes against the raw scores on P3 amplitudes it became evident that the association with SSD was highly nonlinear. We opted for modeling the relationship with a linear term instead of a spline with a possibly high degree of freedom. A linear function would still capture the overall trend evident in our visualization. With this caveat in mind, our ANOVA type III results indicate that the association between Sequence and P3 amplitudes was significant, *F*(1, 44.17) = 48.33, *p* < 0.001, whereas both the stop outcome and SSD was not significantly associated with P3 amplitude, both *p* > 0.05. The interaction between SSD and stop outcome was significant, and a follow-up Tukey corrected simple slope analysis indicated that the two slopes were significantly different, *t*(376) = −2.59, 95% CI = [−0.59, −0.07], *p* = 0.013. Neither of the slopes for each of the stop outcomes were significantly different from zero, both *p* > 0.05. Considering that the linear term included in our model would probably not capture the full relationship between SSD and P3 amplitudes, we interpret this with caution. It is possible that more advanced models could have provided a better model fit for this relationship. The sensitivity analysis further suggests that the results were sensitive to the random-effect specification indicating that robustness could not be established. From the estimated pseudo-R^2^ per predictor variable (see Supplementary material) our results indicate that Sequence had the strongest association with P3 amplitudes with larger P3 amplitudes as the number of go trials prior to a stop trial increase. The difference between stop outcomes was not significant in our model but should be interpreted with the caveat in mind as it is possible that we did not adequately account for the possible interaction between SSD and stop outcome on P3 amplitudes which could bias the estimated difference (see Figure 4).

**Figure 4:**
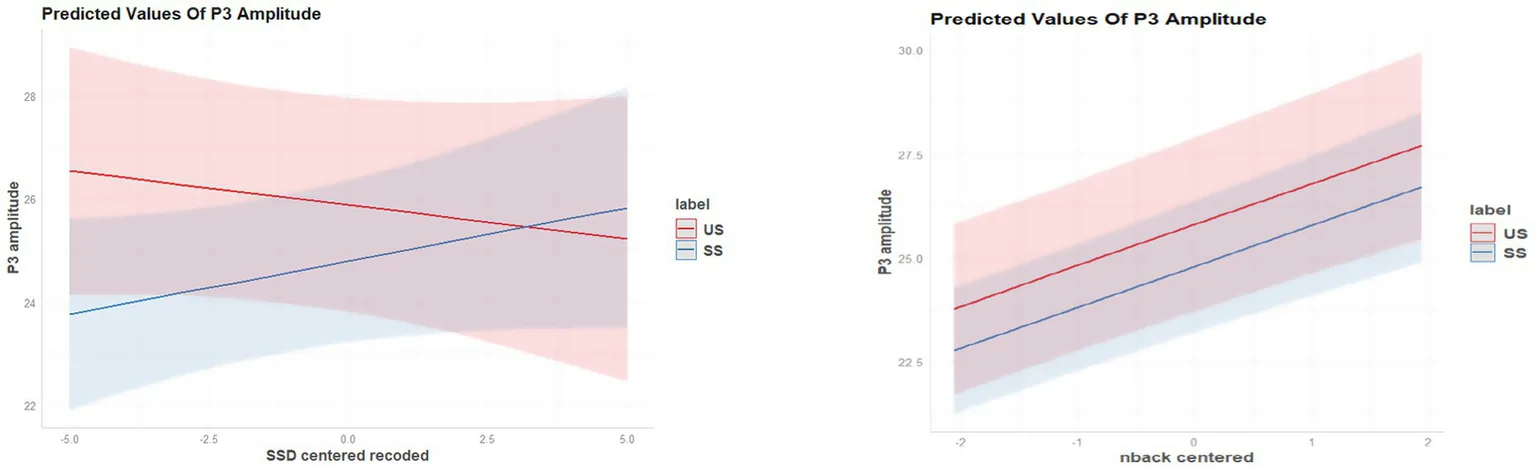
Predicted slopes for SSD and sequence as a function of SS or US. The linear association between SSD and P3 amplitude was highly overlapping between stop outcomes. It is possible that a more complex and nonlinear model could have provided a better understanding of this association. The linear relationship between Sequence and P3 amplitudes was similar across stop outcomes with increasing P3 amplitudes as the number of go-trials increased prior to the stop trial. The model coefficients and the figure indicate an increase of approximately 1 μV per go-trial prior to the stop trial regardless of stop outcome.

## Discussion

Our results show that both sequential dependencies and SSD are differently associated with systematic changes in N2 and P3 component latencies and amplitudes during stop trials in the visual SST. The latencies of both the N2 and P3 were significantly slower during unsuccessful stopping relative to successful stopping, a result that aligns with past research which has indicated delayed neural processing when stopping fails (Huster et al., 2020; Wessel and Aron, 2015; Waller et al., 2021). The predictor variable SSD showed complex curvilinear associations with N2 and P3 latencies where each ERP occurred earlier with longer SSDs. Longer SSDs were associated with faster time-locked neural responses to stop signals. For N2 latency, larger differences between failed and successful stopping were identified near the upper and lower edges of the SSD distribution. In other words, short and long SSDs were associated with larger differences in N2 latency between stop outcomes. For P3 latency, the effect of SSD was similar for both successful and unsuccessful stop trials. SSD also showed a curvilinear relationship with N2 amplitude. The most negative N2 amplitudes coincided with approximately the average SSD values while N2 amplitudes were more positive near the upper and lower edges of the SSD distribution. This pattern of results suggests that sampling the extreme ends of the SSD distribution significantly influenced the difference between stop outcomes for N2 latency, while the N2 amplitude and P3 latency did not show a different scaling between stop outcomes. In contrast to SSD, the effects of sequential dependencies were most prominent in the amplitudes of the N2 and P3. The N2 amplitude was more negative, and the P3 amplitude was more positive with an increasing number of go trials preceding a stop trial. This effect was different for unsuccessful and successful stopping for the N2 amplitude only, where successful stopping was associated with a steeper decrease of amplitude with more preceding go-trials. The P3 amplitude increased at a similar rate for both stopping outcomes, albeit the result from the P3 amplitude analysis should be interpreted with caution as our analysis suggested that more complex relationships might have possibly provided a better model fit. In sum, our mixed effect analysis demonstrates significant and complex associations of both SSD and sequential dependencies on commonly explored EEG indices time-locked to failed and successful stopping at single trial level.

### Sequential dependencies

Only the N2 amplitude significantly covaried with sequential dependencies and further showed a significant interaction between sequence and stop outcome. A longer stretch of go trials made the N2 more negative and this effect was larger when participants succeeded at stopping. No significant effect of sequential dependencies was found on N2 latency. Previous studies investigating trial probabilities on EEG components have mostly focused on changing the global base rate of trials in the SST or in Go/No-Go tasks (Dimoska and Johnstone, 2008; Lavric et al., 2004; Harper et al., 2016), which does not disentangle how sequential dependencies influence EEG indices locally. The linear effect of sequential dependencies on N2 amplitudes indicates that local trial-wise histories influence N2 amplitudes systematically when local trial-histories comprise longer preceding go sequences (including four or more go trials) as well as the immediately preceding stop trial relative to the current stop trial. This is important as larger differences in N2 amplitudes between stop outcomes were observed whenever a stop trial was recently encountered. Past research investigating the behavioral consequences of stopping on the upcoming trials show that the SSRT decreases after a stop trial (Bissett and Logan, 2012a, 2012b). We observed that the N2 amplitude was smaller (meaning less negative) between stop outcomes following a recent stop trial in the local trial-history. This result is interesting considering previous research showing that the N2 amplitude is modulated by sequence-congruency effects (Adelhöfer and Beste, 2020; Suzuki, 2021). In both studies, a significant “current vs previous trial” interaction was present whereby the N2 amplitude was smaller when the previous trial was incongruent relative to congruent. Thus, the N2 amplitude during trials involving conflict is at least sensitive to past trial sequences modulating the difference between congruent and incongruent trials. Having recently experienced an incongruent trial reduced the congruency effect and the same effects were shown on the N2 amplitude (Adelhöfer and Beste, 2020; Suzuki, 2021). Together, this suggests that the N2 amplitude is sensitive to ongoing adaptations comprising the history of past trial sequences which are likely to some extent reflect expectations about what the upcoming trial would be.

Our analysis further shows that in addition to the N2 amplitude, the P3 amplitude was also significantly associated with sequential dependencies. P3 amplitudes scaled linearly with an increasing number of go trials preceding a stop trial (~1 μV per go-trial), and in contrast to N2 amplitudes, this increase was consistent across stop outcomes. Past research has tried to disentangle the effects of inhibition from the effects of novelty by including an ignore-trial type in addition to stop trials (Dimoska and Johnstone, 2008; Waller et al., 2021). These studies found evidence supporting that base rate differences influenced the P3 amplitude further suggesting that the P3 amplitude at least partially reflects behavioral adjustments to salient events during stopping in the SST (Dimoska and Johnstone, 2008; Waller et al., 2021). Interestingly, the effect of base rate was still present in the P3 after having experimentally controlled for differences due to SSDs, aligning with our results on the P3 amplitude (Waller et al., 2021). Given the oddball nature of the SST, these results suggest that a proportion of the variation in P3 amplitude effects between successful and unsuccessful stopping are possibly driven by sequential dependencies. Consequently, accounting for effects of sequential dependencies on the N2 and P3 amplitude is important in future research, particularly in studies focusing on differences between participants sampled from healthy and clinical groups. Important amplitude variation between stop outcomes may reside in how the groups are influenced by sequential dependencies over local trial histories. By including longer preceding go sequences, including sequences of four or more trials, our analysis compliments past research to accommodate sequential dependencies developing across longer trial sequences. These results contribute important evidence to better understand the effects of local dependencies on the N2 and P3 ERPs time-locked to stop stimuli in the SST.

### SSD

The N2, P3 latency as well as N2 amplitude were best modeled by nonlinear relationships with SSD. These results indicate that it is not sufficient to expect a monotonic increase/decrease in N2 or P3 component activity with increasing SSDs (as the difficulty of stopping gets harder). SSD is a complicated variable influenced by past stop outcomes. It makes the task of predicting the onset of the stop signal temporally less palpable and putatively adapts to the duration of the stopping process (Lin et al., 2026). Because SSD is an adaptive task parameter rather than an experimentally controlled variable, current values reflect recent stopping history and possibly the state of the algorithm. Therefore, the observed associations between SSD and ERP signals should be interpreted as reflecting the combined influence of stop signal timing and recent performance, rather than the independent effect of stop signal delay per se. Our analysis suggests that treating SSD as a simple linear confounder may not remove residual variability associated with its effects on brain activity differences. Few studies that we know of have investigated the influence of absolute SSD values on brain activity, but one study that compared BOLD responses between short and long SSDs during successful stopping demonstrated that short SSDs engaged a more widespread network of brain regions than longer SSDs (Lin et al., 2026). Absolute differences in SSD levels engaged similar networks, with short SSDs producing a higher level of activity possibly reflecting increased conflict between going and stopping (Lin et al., 2026). In our analysis, both the N2 and P3 latency peaked later at short SSDs which may be associated with an increased need to resolve conflict by a widespread reconfiguration of networks active during stopping. The significant interaction and curvilinear relationship between N2 latency and stop outcome further suggests that it peaked later at longer SSDs when stopping failed. One possible interpretation of this effect is that the window for more widespread reconfigurations is often too narrow at longer SSDs. At the upper end of SSDs, successful stopping requires faster and less widespread activity.

Results from our modeling further suggest that when participants fail to stop, their neural responses are approximately 20-25 ms slower (the N2 difference is slightly faster relative to the P3 difference). It would be interesting to see whether this approximation could be observed in separate analyses using the onset of partial EMG responses to investigate the relative timing difference of the same contrast (SS vs. US). This could offer insight into the trial-by-trial processing times. Important individual variation may reside in the timing difference rather than the average stopping time (i.e., SSRT). The average stopping time reflects the combination of several parameters and may thus show lower associations with trial-by-trial fluctuations of specific brain activity markers. N2 and P3 latency possessed large inter-trial variation which is what motivated our latency minimization procedure in the first place.

Across our models, intra-individual variation explained a much larger proportion of the variability than the marginal effects alone. One interpretation of this is that our models better captured individual-specific variation in the relationship between predictor variables and dependent variables. For instance, it is possible that the N2 amplitude changed in systematic ways when SSD was increasing for a particular individual while the effect of changing the SSD on N2 amplitudes was much less consistent across individuals. Our analysis focused on between-level effects and cannot answer why or whether this is the case as this would require analyses that target specifically intra-individual variation. However, the ratio between conditional and marginal variability underscores the importance of accounting for the fact that trials are structured within individuals when assessing associations between task parameters and brain activity at single trial level. It is possible that latent modeling of the underlying processes influenced by sequential dependencies and SSD may improve analyses targeting how trial-by-trial variation in EEG components covary with such parameters. For example, if sequential dependencies shape expectations about upcoming trials, latent modelling capturing how individuals form subjective probabilities can improve analyses linking trial-by-trial changes in probabilities to EEG components. Future research could investigate the mechanisms underlying the influence of SSD and sequence effects on the relative time or amplitude difference between stop outcomes and include an assessment of whether the strength of the association differs between groups in larger samples.

### Group effects in clinical studies

D'Alberto et al. (2018) found evidence demonstrating that accounting for absolute SSD levels experienced by participants reduced brain activity group differences in key networks commonly associated with performance during the stop signal task. Our analysis extends on these findings by showing that commonly estimated stop-locked EEG components share complex relationships with SSD indicating the need for evaluating the shape of the covariate in analyses aiming to control for SSD differences. Together, these results emphasize the necessity to account for SSD distributions when contrasting successful and failed stopping between groups. If systematic differences in SSD distributions exist, accounting for these differences may lead to less biased estimates of group differences. Furthermore, differences may also arise in the effects of sequential dependencies on brain activity and could, like SSD, be accounted for by including this as a covariate in analyses studying group effects using the SST.

## Conclusion

The results of this study support the relevance of considering both sequential dependencies and SSD when contrasting successful and failed stopping using EEG. Modeling both predictor variables is particularly important to consider when comparing groups using the SST due to the typically implemented staircase design of the SSD and the large inter-individual variability. Failing to account for this variation in the ERP estimates may produce biased estimates of group differences, particularly whenever systematic differences exist in the SSD distributions between the groups being compared. A careful inspection of the shape of modeled relationships is further motivated by our analysis as nonlinear relationships were found to characterize the associations with SSD. In sum, by showing that amplitudes and latencies of the N2 and P3 share complex and unique relationships with task parameters in the SST, our results contribute important knowledge into understanding trial-by-trial variation in neural processes during stopping.

## Data Availability

The raw data supporting the conclusions of this article will be made available by the authors, without undue reservation.
