# Supplementary material for: Sequence effects and SSD are associated with N2 and P3 signals during stopping in a visual stop signal task
Source: Front Hum Neurosci. 2026 Oct 12;20:1906495. doi: 10.3389/fnhum.2026.1906495 (PMC13696411; doi:10.3389/fnhum.2026.1906495)
Supplement: Supplementary file 1 [file Data_Sheet_1.PDF]

Supplementary material

Table of contents

1 Behavioral descriptives 1
2 Description of method for latency minimization 3
3 Statistical analyses 6
    3.1 Dependent variable: N2 latency 6
    3.2 Dependent variable: N2 amplitude 10
    3.3 Dependent variable: P3 latency 13
    3.4 Dependent variable: P3 amplitude 15
4 Simulation analysis for peak extraction procedure 18
5 Electrode layout 32 channels acticap 22
6 Psychtoolbox VBLSyncTest results 23
7 Sensitivity analysis 23

1 Behavioral descriptives

We extracted the raw reaction times for all go trials and all unsuccessful stop trials per participant. For each of these variables, we estimated the mean and the standard deviation. We also extracted the difference between reaction times in unsuccessful stop trials relative to the SSD value.

Figure 1

The first histogram shows the average reaction time for go trials per participant, and the second histogram shows the corresponding standard deviation.

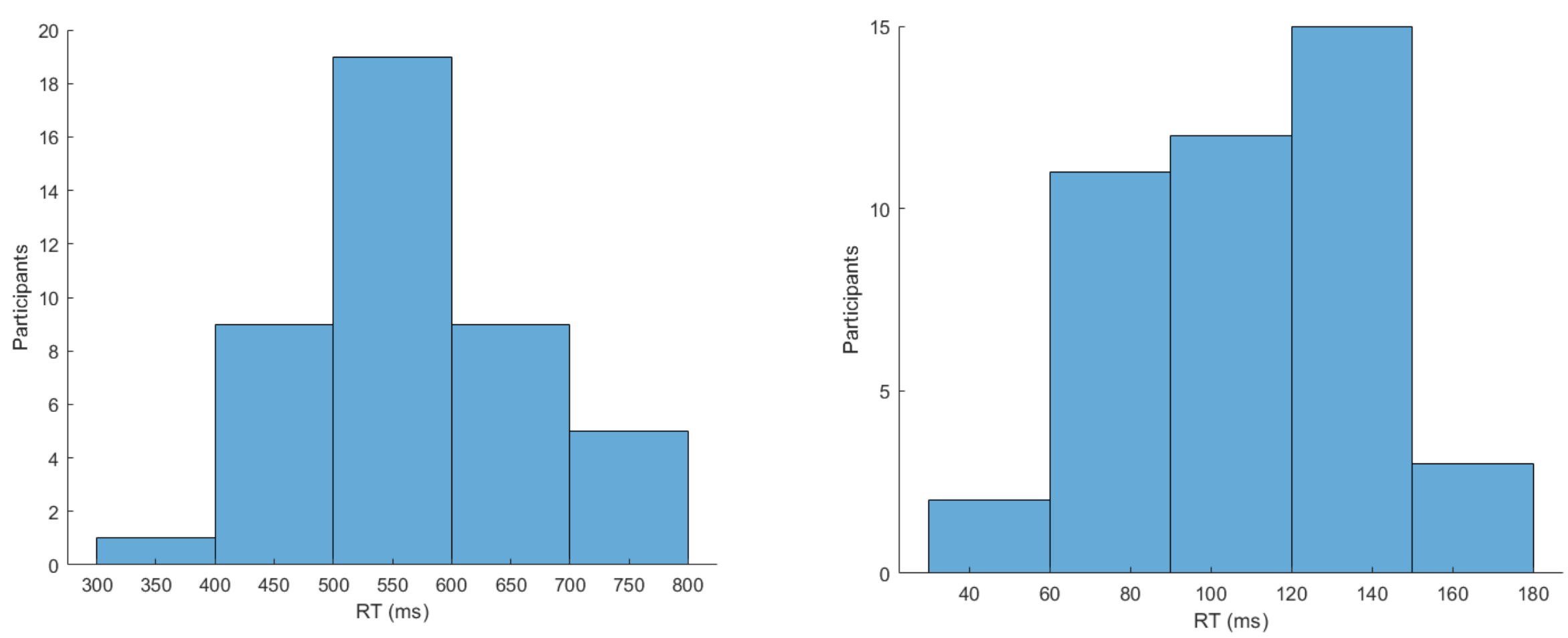

Note. The grand mean and standard deviation across all participants' mean and standard deviation was 560.32ms and 109.48ms.

Figure 2

The first histogram shows the average reaction time for unsuccessful stop trials per participant, and the second histogram shows the corresponding standard deviation.

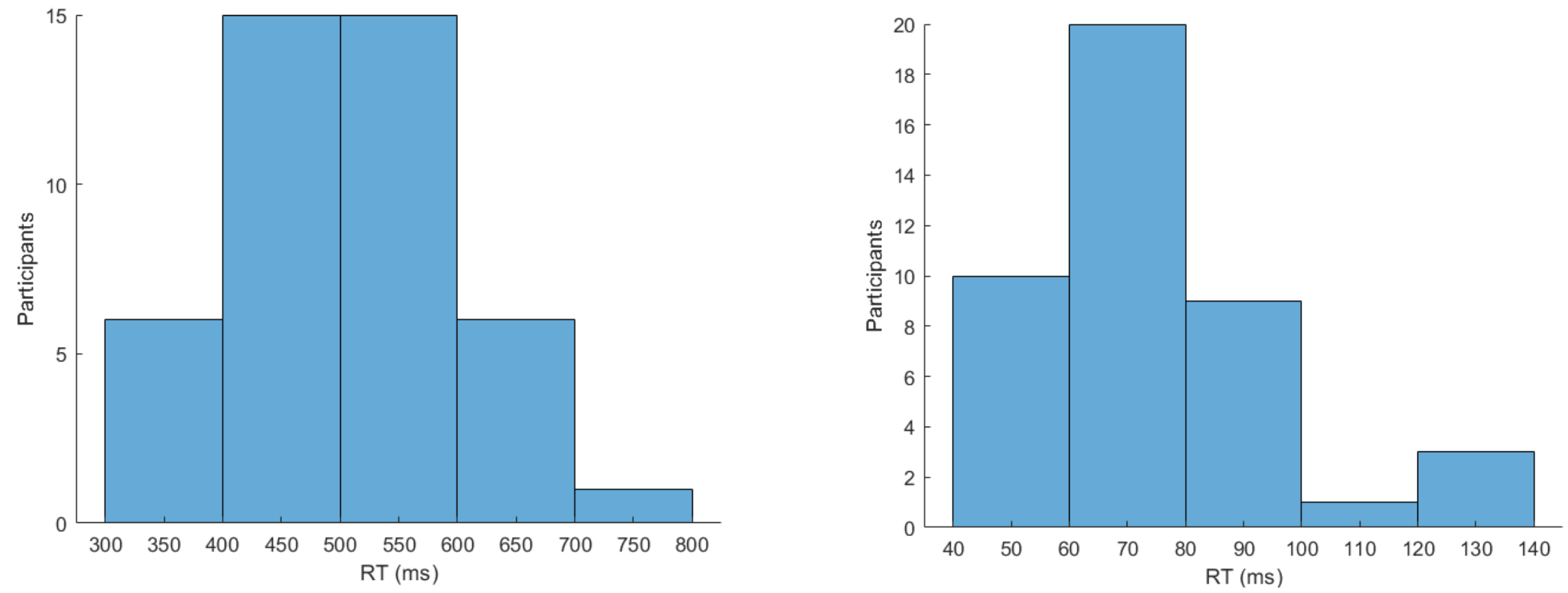

Note. The grand mean and standard deviation across all participants' mean and standard deviation was 511.08ms and 74.10ms.

Across all participants, the average reaction time difference between unsuccessful and go trials is roughly 50ms.

Figure 3

The first histogram shows the single trial reaction time for go trials and the second histogram shows the reaction time for unsuccessful stop trials across participants

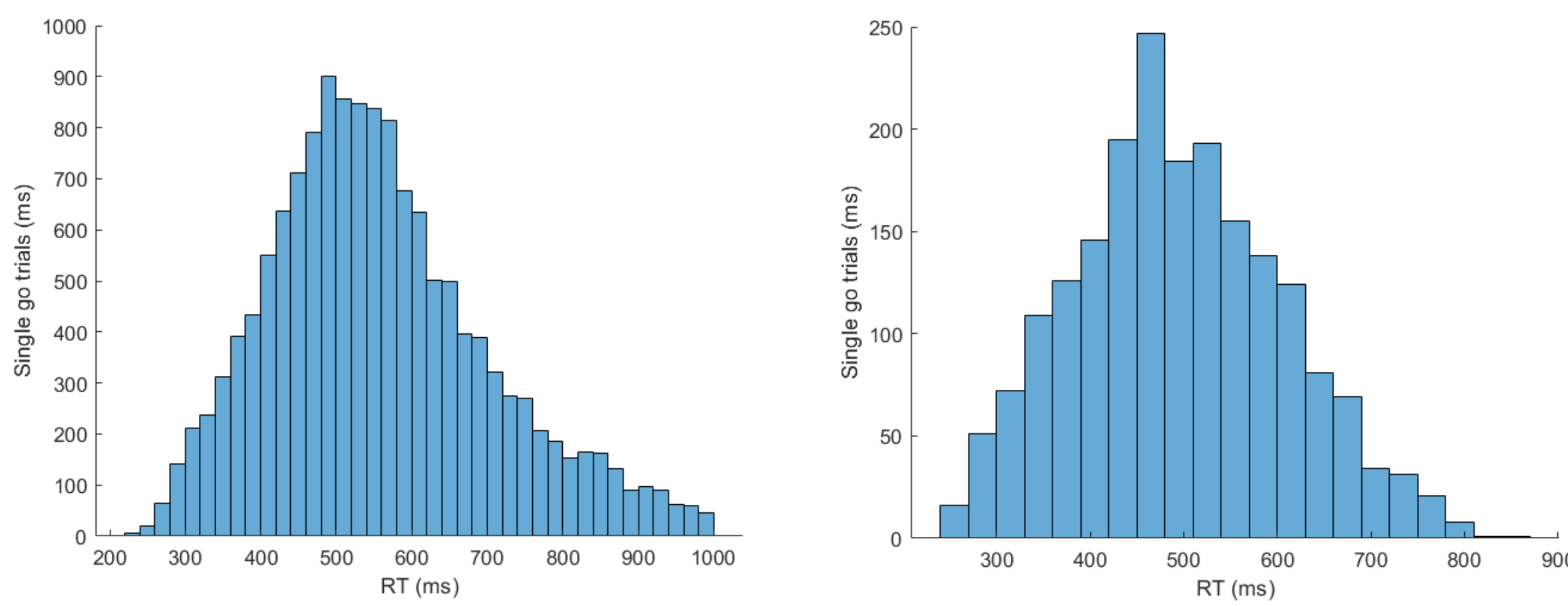

Note. The average go reaction time was 557.14ms, std = 146.31ms. The average reaction time for unsuccessful stop trials was 494.32ms, std = 112.14ms.

Figure 4

Unsuccessful reaction times minus SSD per trial across participants

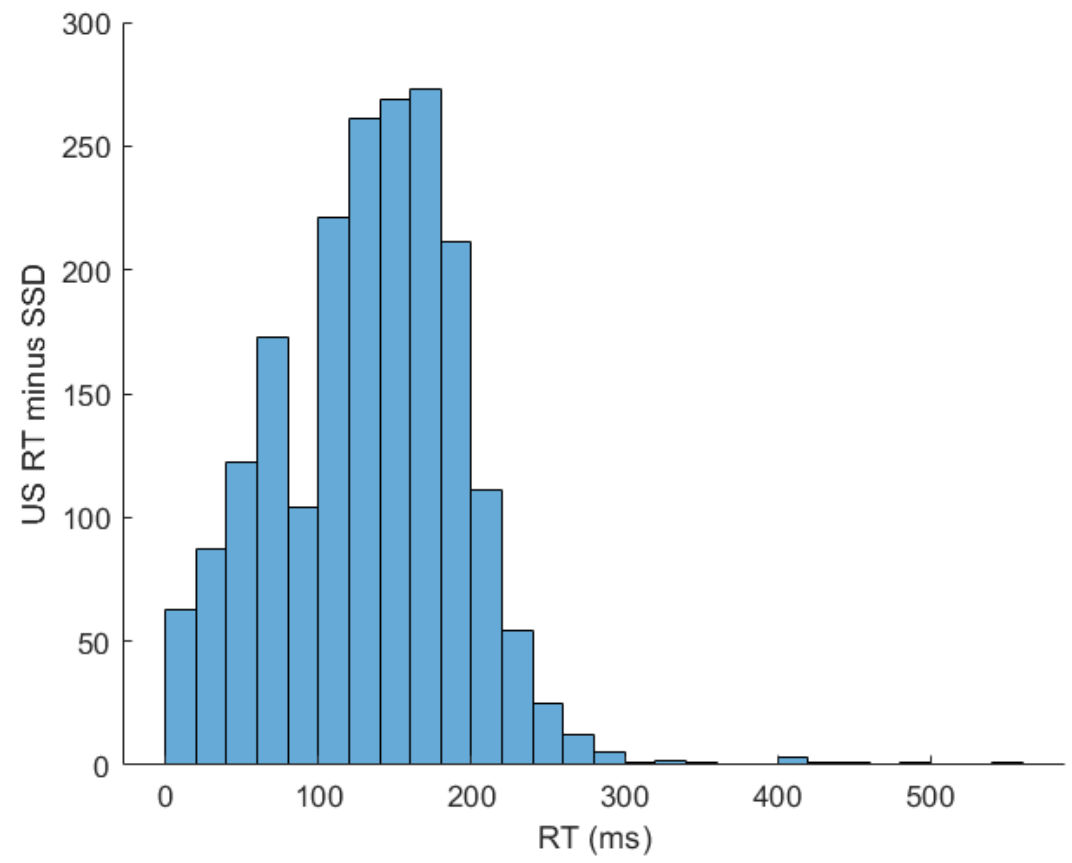

Note. The average difference was 113.06ms, std = 61.63ms.

2 Method used to identify peaks in single trial continuous EEG data

The problem is that single-trial evoked potentials to stimulus onsets can vary in time making peak detection difficult. This variation can be minimized by temporally shifting single-trials relative to each other. Because no reference signal is available, the method stops when the difference between the average ERP from the preceding iteration is negligible. Due to the 1/f characteristic of EEG and that most of the variability in N2 and P3 components at Cz resides in the lower frequency range, the continuous EEG data was high-pass filtered at 1.5Hz and low-pass filtered at 12Hz. Initial guesses for onset time points were stop signal onsets. The method has two loops. The outer loop is responsible for penalizing large shifts and controlling when the difference in ERP is negligible relative to the last iteration. The inner loop finds the optimal lags to shift each single trial to minimize their difference. The whole method is detailed in steps below. The inner loop involves steps 1:10 while the outer loop involves steps 11:13.

Step 1 Limit search area to defined time interval. Hann taper and z-score each trial. Taper length is the length of each trial. The effect is to reduce extreme edge values.

Step 2 Hann taper each single trial. The taper is centered at 250ms and zero-padded before -300ms and after 800ms relative to time point zero.

Step 3 For each single trial: calculate the normalized cross-correlation with all the single trials. This produces a matrix A comprising the estimated lag in ms between all single trials.

Step 4 For each single trial, calculate the 5th percentile based on cross-correlation from step 3. Extract the trials which have distances below the percentile. Loop over these extracted trials and calculate the cross-correlation against the single trial. Shift the extracted trials to match the single trial. Concatenate the single trial with the shifted trials and calculate the average. Store the average for each single trial in matrix B.

Step 5 Take the mean across all trials in matrix B. Z transform and taper with window defined in step 2. This is the new template.

Step 6 Taper each single trial in matrix B with the window from step 2.

Step 7 Loop over trials in matrix B from step 6. Cross-correlate between the new template and each single trial. Calculate the lag. If the lag is beyond +-30, compute the Euclidian distance by shifting the trial from -20:20 sampling points. The minimum distance yields the best lag. Retain all lags.

Step 8 Shift the single trial original data with the lags.

Step 9 Calculate the ERP of the shifted data.

Step 10 Calculate the mean-square error (MSE) between the ERP of the shifted data and the original ERP without shifting.

Step 11 Reduce the impact of large lags. This is defined as lags above 30ms by multiplying these lags by 0.7.

Step 12 Epoch the unshifted data again with the new lags (new time point zero).

Step 13 Calculate the difference between the current MSE and the previous MSE. Stop the iteration if the change from previous iteration is below 0.01 - representing a negligible change. Alternatively, stop the iterations if any change from initial time-point 0 is above 400ms or below each trials SSD multiplied with 0.3.

The outcome of the algorithm is a narrower window size for which to detect single trial peaks. We calculated the difference between the maximum and minimum latency of the minimized and the unshifted dataset. Across 43 participants, the average percentage decrease in window size was ~34% meaning that the window for which the N2 and P3 peaked was reduced following latency minimization.

Figure 5

Stop stimulus locked ERPs from an example participant before (first plot) and after (second plot) minimizing lag differences at single trial. Single trial data has been transformed using z scores and tapered for visualization purposes.

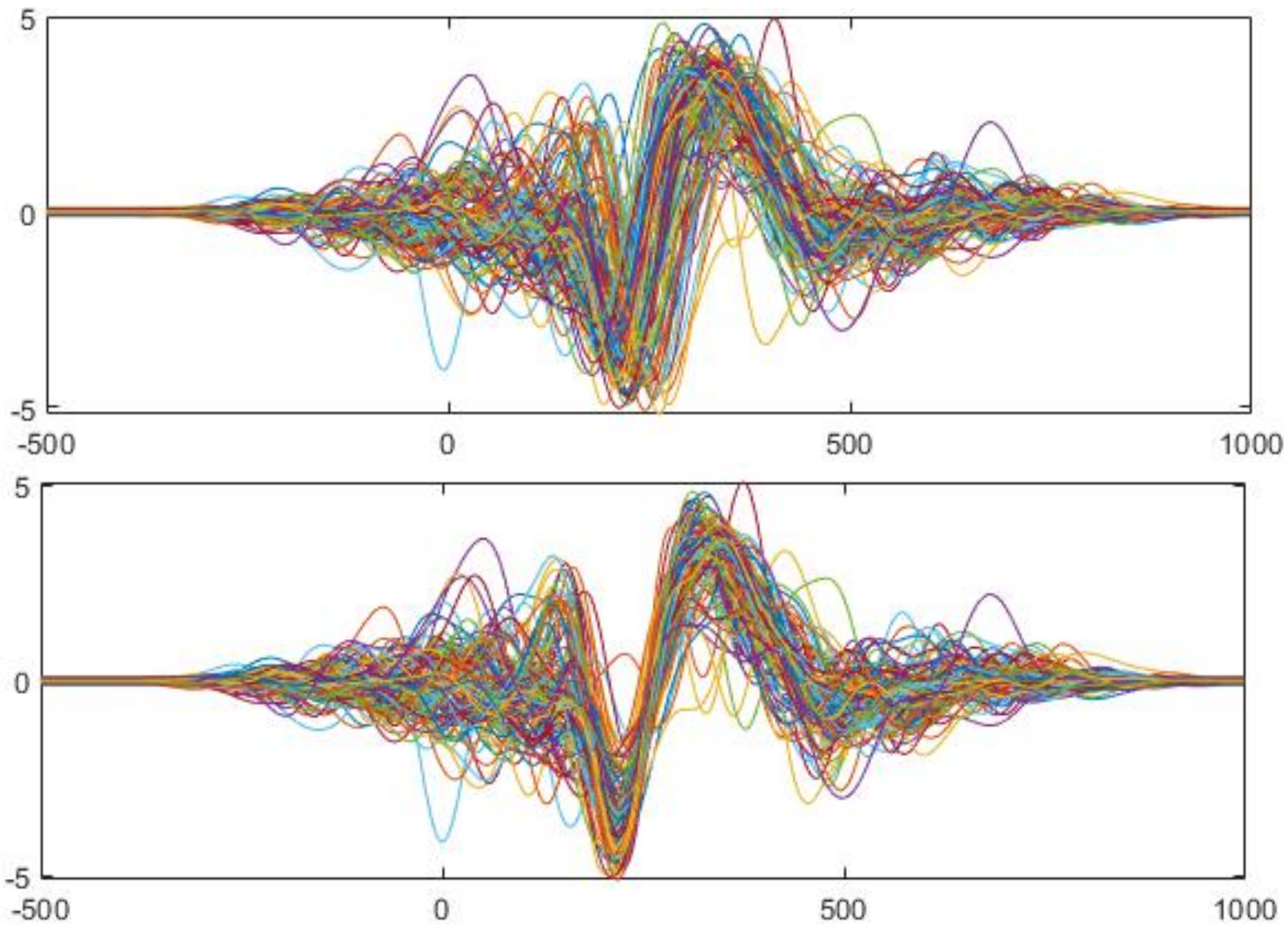

Note. The first plot shows the raw temporal variation in single trial EEG data, and the second plot shows the same single trial data after temporally shifting the single trials. The method has reduced inter-trial temporal variation.

A short note on the experience from making this method. In stop trials, there will always be a go stimulus first (S1) and a stop stimulus second (S2). If the same oscillations were constantly modulated by the same stimulus to S1, and modulated to the same degree, then one should expect that phase angles for those oscillations should cluster closely together across trials. If the same oscillations were constantly modulated by S2, and this happens systematically at specific phase angles modulated by S1, then reducing latency variation would probably not be needed. We did not find that this was the case and the complexity of the patterns observed in time-frequency space left us to proceed in the time-domain where amplitude reflects the summed activity across multiple oscillations.

3 Statistical analysis

3.1 Dependent variable: N2 latency

Figure 6

Probability density function of N2 latencies

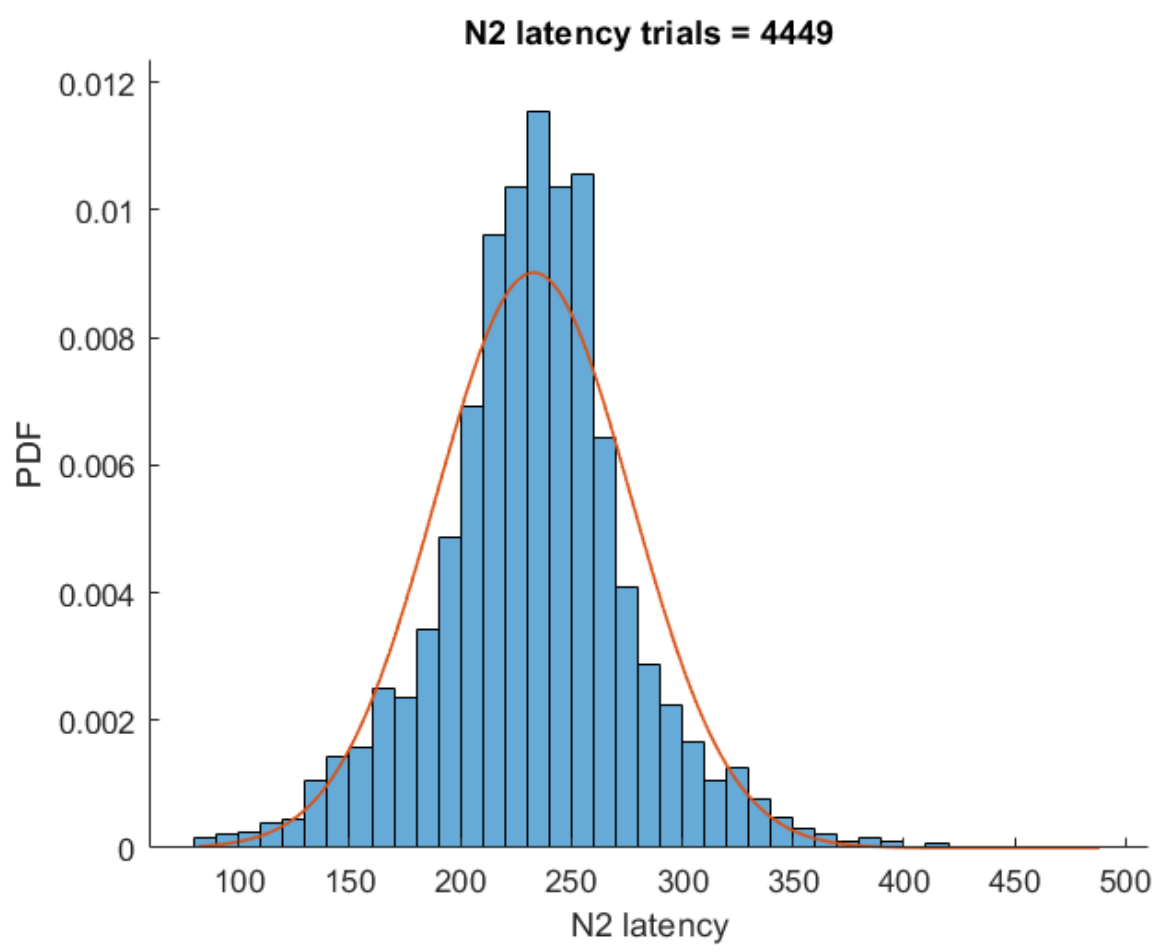

Note. Visual inspection of the raw score distribution and the estimated probability density function indicated a normal distribution.

Intra-class correlation coefficient:

The intra-class correlation coefficient of the null model was ~21%.

Table 1. Model comparisons

	Numpar	AIC	BIC	logLik	-2*log(L)	χ^2	*df*	*p*
Comparison of null Model vs. Model 1								
ModNull	3	44634	44654	-22314	44628			
Mod 1	8	44292	44343	-22138	44276	352.21	5	< 0.001
Comparison of Model 1 vs. Model 2								
Mod 1	8	44292	44343	-22138	44276			
Mod 2	13	44274	44357	-22124	44248	28.246	5	< 0.001
Comparison of Model 2 vs. Model 3								
Mod 2	13	44274	44357	-22124	44248			
Mod 3	17	44275	44383	-22120	44241	7.4107	4	0.1157
Model 2 is selected.								
Comparison of Model 2 vs. Model 4								
Mod 4	10	44280	44343	-22130	44260			
Mod 2	13	44274	44357	-22124	44248	11.694	3	0.0085

Comparison of Model 2 vs. Model 5								
Mod 5	10	44285	44348	-22132	44265			
Mod 2	13	44274	44357	-22124	44248	16.679	3	< 0.001
Comparison of Model 2 vs. Model 6								
Mod 6	10	44283	44347	-22132	44263			
Mod 2	13	44274	44357	-22124	44248	15.373	3	0.0015
Comparison of Model 2 vs. Model 7								
Mod 7	13	44280	44363	-22127	44254			
Mod 2	13	44274	44357	-22124	44248	6.308		

Model 2 is selected.

Results from comparing different random effect structures using likelihood ratio tests indicated that model 2 provided a better model fit. The selected random effect structure:

Model 2: 1 + SSD + Sequence | ID

Visualizing the model:

Plotting the model slopes against scores on the dependent variable suggested that a spline term could improve model fit for the predictor variables SSD and possibly Sequence. A likelihood ratio test indicated that adding splines for SSD provided a better model fit, $\chi(6)$ = 47.35, $p < .001$. Including the interaction for SSD and Group provided a better model fit, $\chi(6)$ = 16.526, $p = 0.011$, so did including the interaction for Sequence and Stop outcome, $\chi(6)$ = 16.821, $p = 0.019$. A likelihood ratio test indicated no statistical difference between a model with and without random slopes for the predictor variable Sequence, $p = 0.05$, so we selected the parsimonious model without random slopes for Sequence. We also tested whether removing random slopes for label provided a better fit, but the likelihood ratio test indicated that a model with random slopes for SSD and Stop outcome provided a better fit, $\chi(3)$ = 10.277, $p = 0.016$. The selected model structure for the final model:

Model: ~ 1 + SSD * Stop outcome + Sequence + (1+ SSD + Stop outcome | ID)

Figure 7

Visualization of residuals

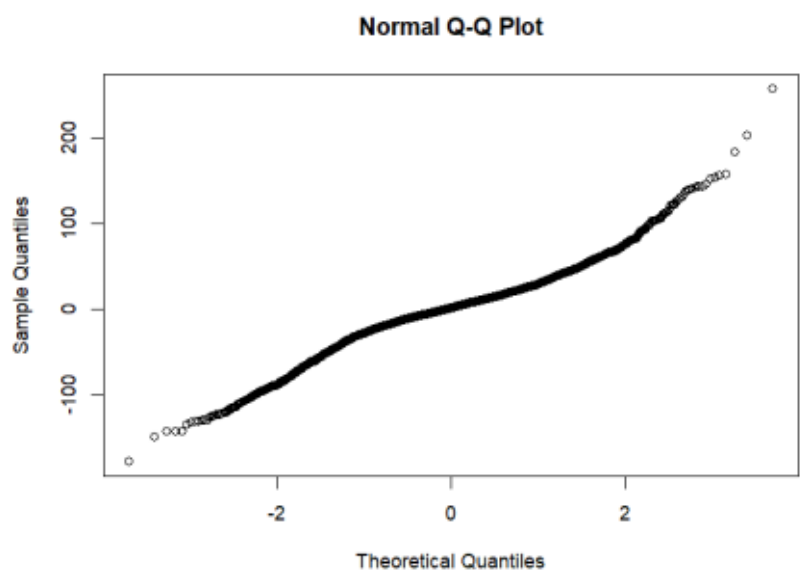

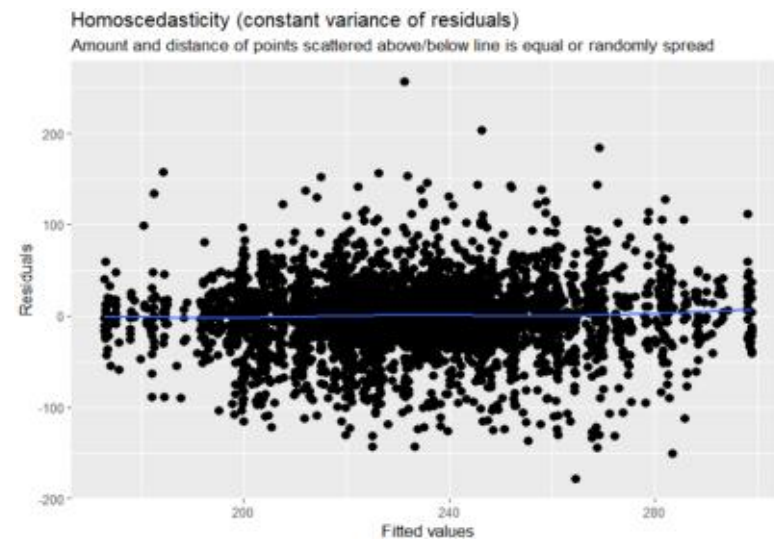

Note. Residuals were visually checked and did not cause any reason for concern. Although there is a slight curvature in the QQ-plot, the linear shape is clearly characterizing the distribution.

Table 2. Standardized beta coefficients

Parameter	Std. Coef.	95% CI
Intercept	0.70	[0.50, 0.90]
SSD sp. 1	-1.48	[-1.80, -1.17]
SSD sp. 2	-0.34	[-0.50, -0.17]
Stop outcome	0.29	[0.18, 0.39]
Sequence	6.33e-03	[-0.02, 0.03]
SSD sp.1 * Stop outcome	-0.09	[-0.30, 0.12]
SSD sp.2 * Stop outcome	0.14	[0.04, 0.24]

Note. Standardization method: refit.

Table 3. Pseudo-R^2 coefficients

	Effect	Rsq	95% CI
1	Model	0.103	[0.088, 0.121]
2	SSD	0.047	[0.035, 0.059]
3	Stop outcome	0.007	[0.003, 0.013]
4	SSD * Stop outcome	0.002	[0.006, 0]
	Sequence	0	[0.001, 0]

Note. Estimated marginal and conditional R^2: ~10% and ~27%.

Follow-up testing of significant interaction between SSD and Stop outcome

Since the predictor variable SSD comprised 11 levels, the difference between mean values for each level were statistically compared with Bonferroni correction for multiple comparison. Larger mean differences are observed for extreme ends of the SSD variable. Output from R studio can be seen below.

Table 4. t-test of mean difference at each level of the predictor variable SSD

**Level**	**Estimate**	**SE**	**df**	**t-ratio**	**p**

SSD = -5	25.3	4.62	167.5	5.479	<.001
SSD = -4	22.9	3.40	129.5	6.741	<.001
SSD = -3	20.7	2.44	85.8	8.517	<.001
SSD = -2	19.1	1.93	60.8	9.872	<.001
SSD = -1	18.2	1.89	65	9.628	<.001
SSD = 0	18.3	1.97	76.2	9.288	<.001
SSD = 1	19.6	1.93	73.2	10.127	<.001
SSD = 2	21.8	1.88	65.6	11.584	<.001
SSD = 3	24.8	2.13	78.6	11.687	<.001
SSD = 4	28.4	2.80	119.8	10.136	<.001
SSD = 5	32.1	3.77	166.6	8.525	<.001

Note. Contrast = US – SS, Satterthwaite degrees of freedom. Bonferroni-corrected.

Figure 8

Mean differences between US and SS for each SSD level

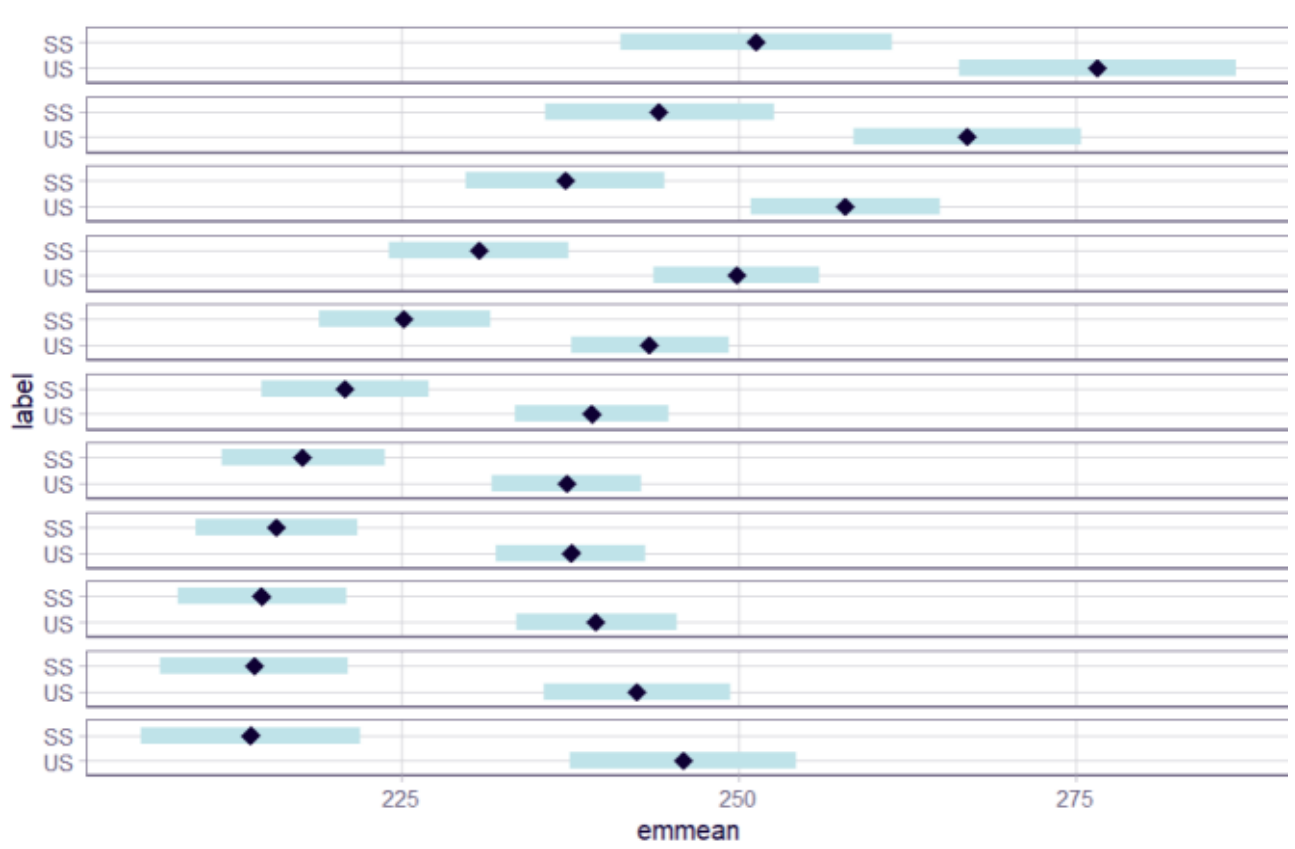

3.2 Dependent variable: N2 amplitude

Figure 9

Probability density function of transformed N2 amplitude

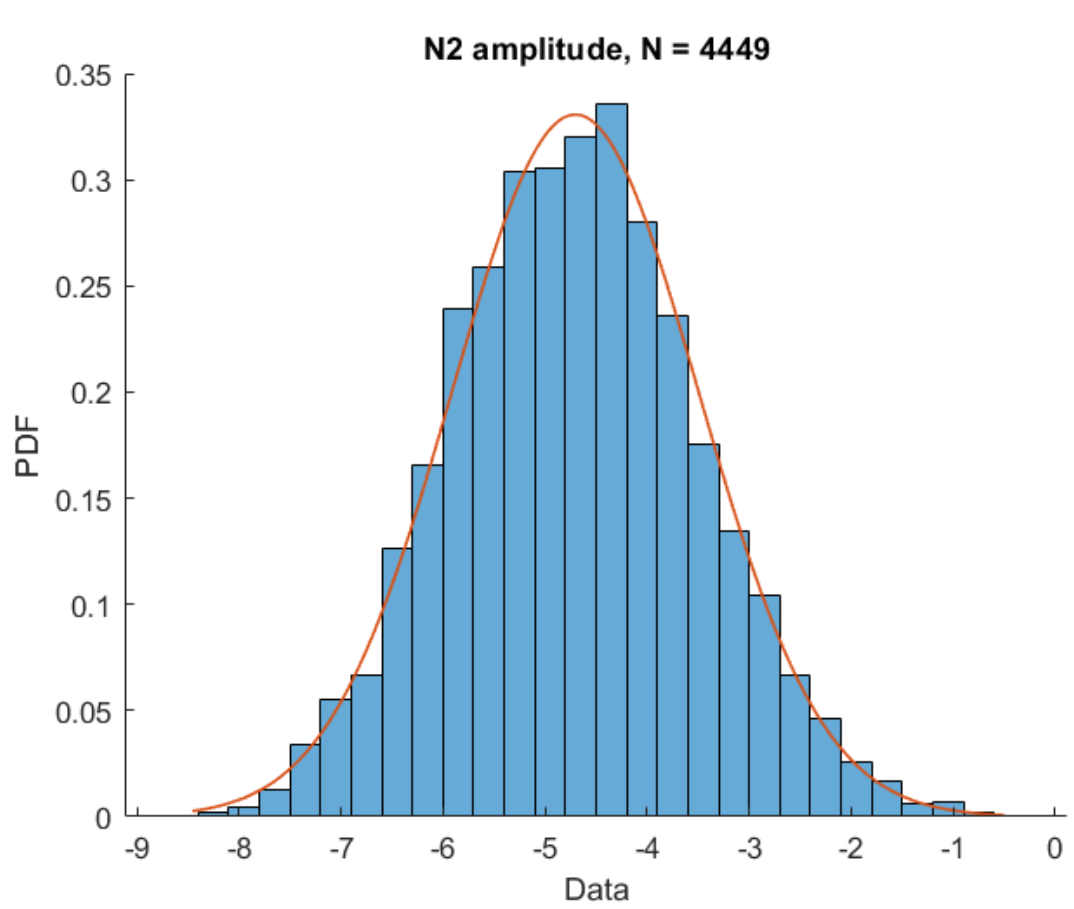

Note. We visually inspected the distribution of raw N2 amplitude scores. Because the distribution looked slightly skewed, we transformed the variable using a square root transformation of the absolute values and multiplied with -1 to make the values negative (for interpretation).

Intra-class correlation coefficient

The intra-class correlation coefficient estimated from the null model was ~29%.

Table 5. Model comparisons.

	**Numpar**	**AIC**	**BIC**	**logLik**	**-2*log(L)**	χ^2	***df***	***p***
Comparison of null Model vs. Model 1								
ModNull	3	12740	12759	-6366.7	12734			
Mod 1	8	12287	12338	-6135.6	12271	462.37	5	< 0.001
Comparison of Model 1 vs. Model 2								
Mod 1	8	12287	12338	-6135.6	12271			
Mod 2	13	12207	12290	-6090.3	12181	90.498	5	< 0.001
Comparison of Model 2 vs. Model 3								
Mod 2	13	12207	12290	-6090.3	12181			
Mod 3	17	12116	12225	-6041.1	12082	98.502	4	< 0.001
Comparison of Model 3 vs. Model 4								
Mod 4	10	12210	12274	-6095.1	12190			
Mod 3	17	12116	12225	-6041.1	12082	108.03	7	< 0.001
Comparison of Model 3 vs. Model 5								
Mod 5	10	12282	12346	-6131.2	12262			
Mod 3	17	12116	12225	-6041.1	12082	180.38	7	< 0.001
Comparison of Model 3 vs. Model 6								
Mod 6	10	12194	12258	-6087.1	12174			
Mod 3	17	12116	12225	-6041.1	12082	92.178	7	< 0.001
Comparison of Model 3 vs. Model 7								
Mod 7	13	12198	12282	-6086.3	12172			
Mod 3	17	12116	12225	-6041.1	12082	90.413	4	< 0.001

Results from comparing different random effect structures using likelihood ratio tests indicated that model 3 provided a better model fit. The selected random effect structure:

Model 3: 1 + SSD + Sequence + label | ID

Visualizing the model:

Plotting the model slopes against scores suggested that a spline term could improve model fit for the predictor variable SSD. A likelihood ratio test indicated that adding splines for SSD provided a better model fit, $\chi(2) = 55.65$, $p < .001$. Including the interaction for SSD and Stop outcome did not provide a better model fit, $\chi(2) = 1.861$, $p = 0.394$, whereas a model including the interaction for Sequence and Stop outcome was better, $\chi(1) = 8.961$, $p = 0.002$. A likelihood ratio test indicated that including random slopes for SSD provided a better model fit, $\chi(4) = 52.583$, $p < .001$, whereas excluding random slopes for Sequence did not significantly provide a better fit, $\chi(4) = 2.112$, $p = 0.715$, so we selected the parsimonious model without random slopes for Sequence.

Final model: ~ 1 + SSD + Sequence * Stop outcome + Stop outcome + (1+ SSD + Stop outcome | ID)

Figure 10

The first subplot shows the qq-norm of the residuals, while the second shows the fitted values against the residuals

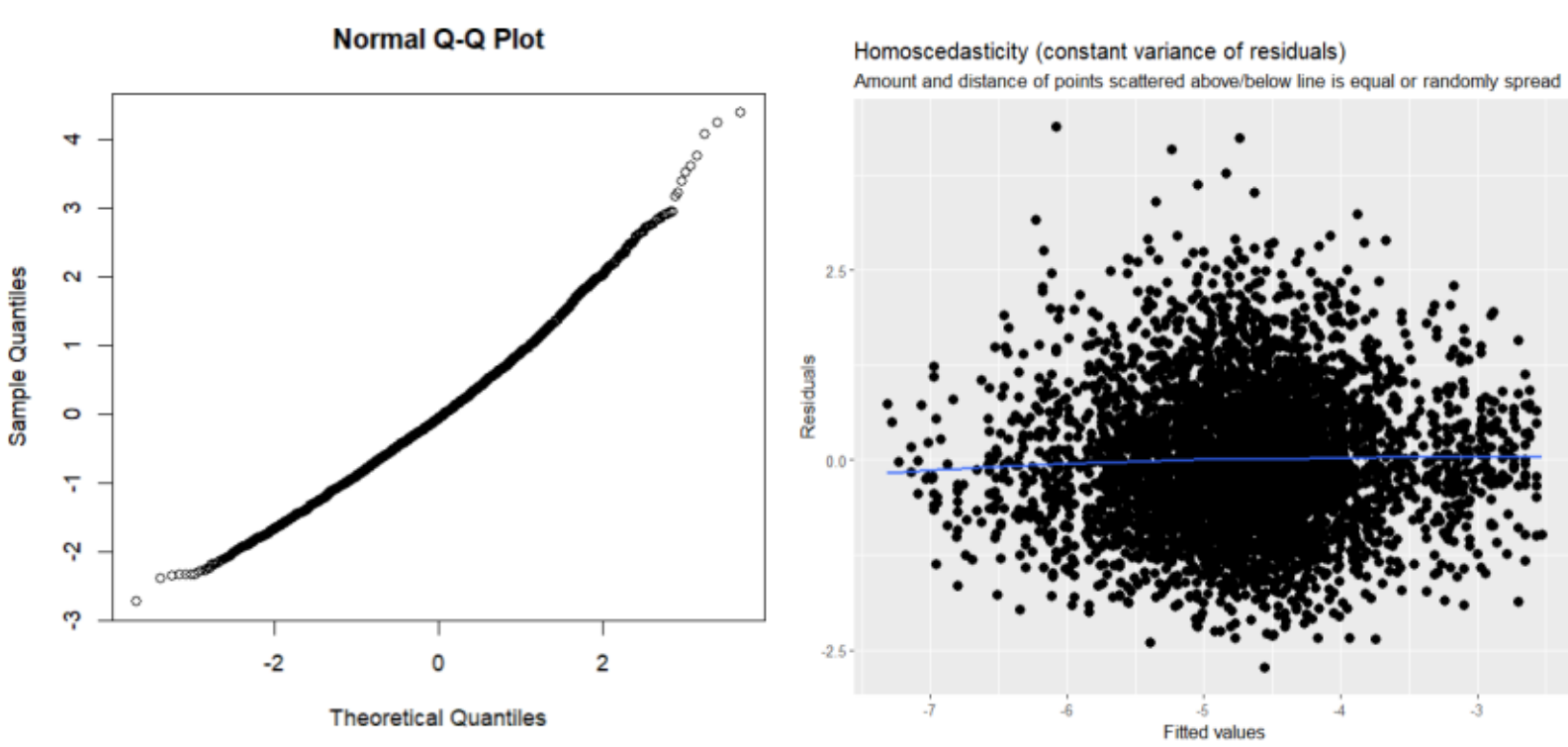

Note. Visual inspection of the residuals gave no reason for concern.

Table 6. Standardized beta coefficients

Parameter	Std. Coef.	95% CI
Intercept	0.66	[0.42, 0.91]
SSD sp. 1	-1.49	[-1.87, -1.10]
SSD sp. 2	-0.08	[-0.31, 0.14]
Stop outcome	-0.21	[-0.27, -0.05]
Sequence	-0.07	[-0.09, -0.05]
Sequence * Stop outcome	0.04	[0.01, 0.06]

Note. Standardization method: refit.

Table 7. Pseudo-R^2 coefficients

	Effect	Rsq	95% CI
1	Model	0.096	[0.081, 0.113]
2	Stop outcome	0.043	[0.032, 0.055]
3	SSD	0.039	[0.029, 0.051]
4	Sequence	0.005	[0.002, 0.010]
	Sequence*Stop outcome	0.001	[0, 0.004]

Note. Estimated marginal and conditional R^2: ~10% and ~45%.

Simple-slope analysis of significant interaction between Sequence and stop outcome

We conducted a simple slope analysis to statistically evaluate whether the association between Sequence and Stop outcome was significantly different between successful and unsuccessful stop trials. A significant difference was observed (Tukey adjusted) whereby the reduction in N2 amplitudes with higher Sequence lengths was larger for successful relative to unsuccessful stop trials.

Table 8. Mean differences with Tukey corrected p-values

Level	Estimate	SE	df	t-ratio	p
US	-0.026	0.013	4282	-1.941	0.052
SS	-0.081	0.013	4299	-6.469	< 0.001
US - SS	0.056	0.018	4286	3.039	0.002

Note. Satterthwaite degrees of freedom. Confidence interval = 0.95.

3.3 Dependent variable: P3 latency

Figure 11

Probability density function of P3 latency

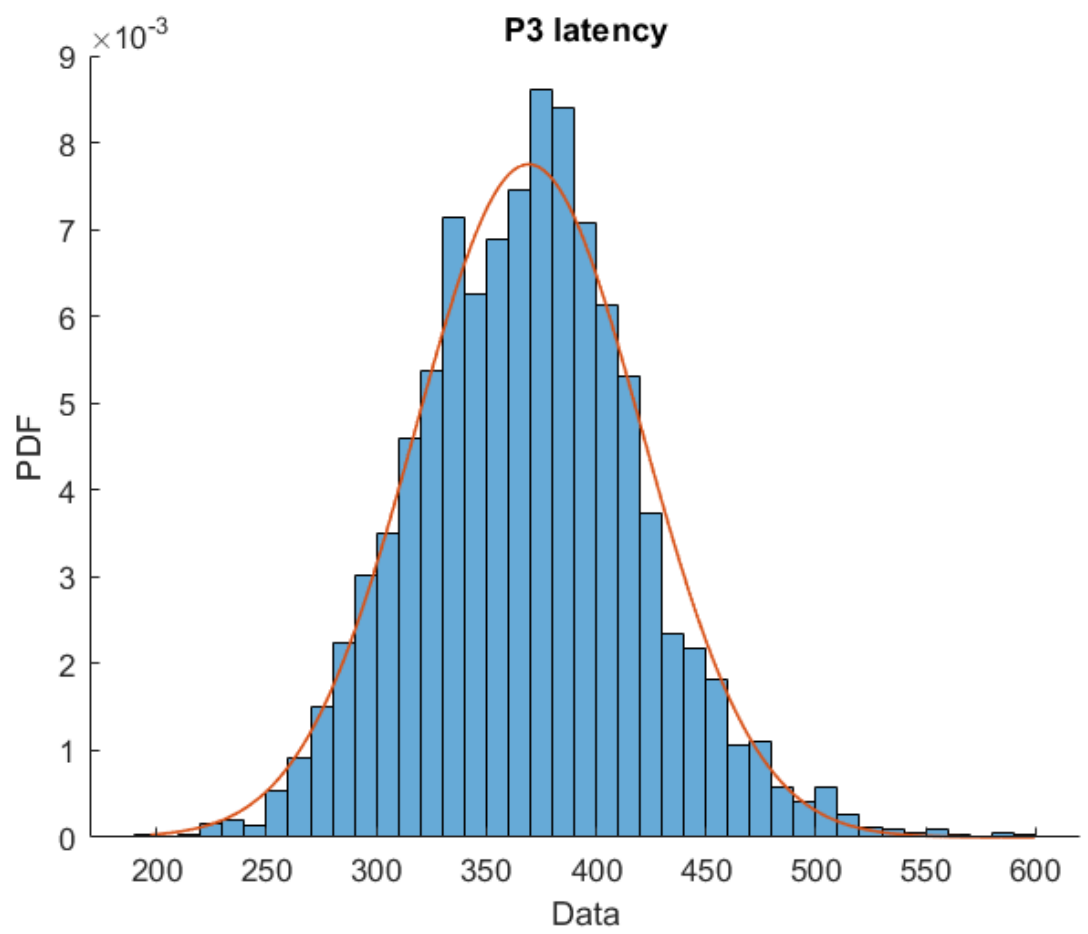

Note. Visual inspection of the raw scores and the fitted density function indicated a normal distribution.

Intra-class correlation coefficient:

The intra-class correlation coefficient of the null model was ~25%.

Table 9. Model comparisons

	**Numpar**	**AIC**	**BIC**	**logLik**	**-2*log(L)**	χ^2	***df***	***p***
Comparison of null Model vs. Model 1								
ModNull	3	46099	46118	-23047	46093			
Mod 1	8	45863	45914	-22923	45847	246.63	5	< 0.001
Comparison of Model 1 vs. Model 2								
Mod 1	8	45863	45914	-22923	45847			
Mod 2	13	45863	45946	-22918	45837	9.878	5	0.078
Model 3 failed to converge – is singular								
Mod 1	8	45863	45914	-22923	45847			
Comparison of Model 1 vs. Model 4								
Mod 1	8	45863	45914	-22923	45847			
Mod 4	10	45858	45922	-22919	45838	8.760	2	0.012
Comparison of Model 4 vs. Model 5 – mod4 has lower AIC and BIC								
Mod 5	10	45866	45929	-22923	45846			
Mod 4	10	45858	45922	-22919	45838	7.725	0	NaN
Comparison of Model 3 vs. Model 6 – mod6 has lower AIC and BIC								
Mod 4	10	45858	45922	-22919	45838			
Mod 6	10	45848	45912	-22914	45828	9.515	0	NaN

Results from comparing different random effect structures using likelihood ratio tests indicated that model 6 provided a better fit. The selected random effect structure:

Model 6: 1 + label | ID

Visualizing the model:

Raw scores against the predicted slopes suggested that nonlinear shapes of both SSD and Sequence could provide a better fit. A likelihood ratio test favored including splines for both SSD and Sequence, $\chi(4) = 23.09$, $p < .001$. We tested whether adding splines only for SSD was favored over a model including splines for both continuous predictor variables. A likelihood ratio test indicated that adding splines for SSD and a linear term for Sequence produced no difference to the more complex model with splines for both terms, $\chi(2) = 5.70$, $p = 0.057$. The simpler model was selected with splines for SSD and a linear term for Sequence. A likelihood ratio test indicated no significant difference between a model without interaction between SSD and Stop outcome, $\chi(2) = 1.11$, $p = 0.574$, or interaction between Sequence and Stop outcome, $\chi(2) = 1.82$, $p = 0.174$. We again checked the random effect structure by comparing models and excluded models resulting in singular fits.

The final model: ~ 1 + SSD + Sequence + Stop outcome + (1+ Stop outcome | ID)

Figure 12

The first subplot shows the QQ-norm of the residuals, while the second shows the fitted values against the residuals

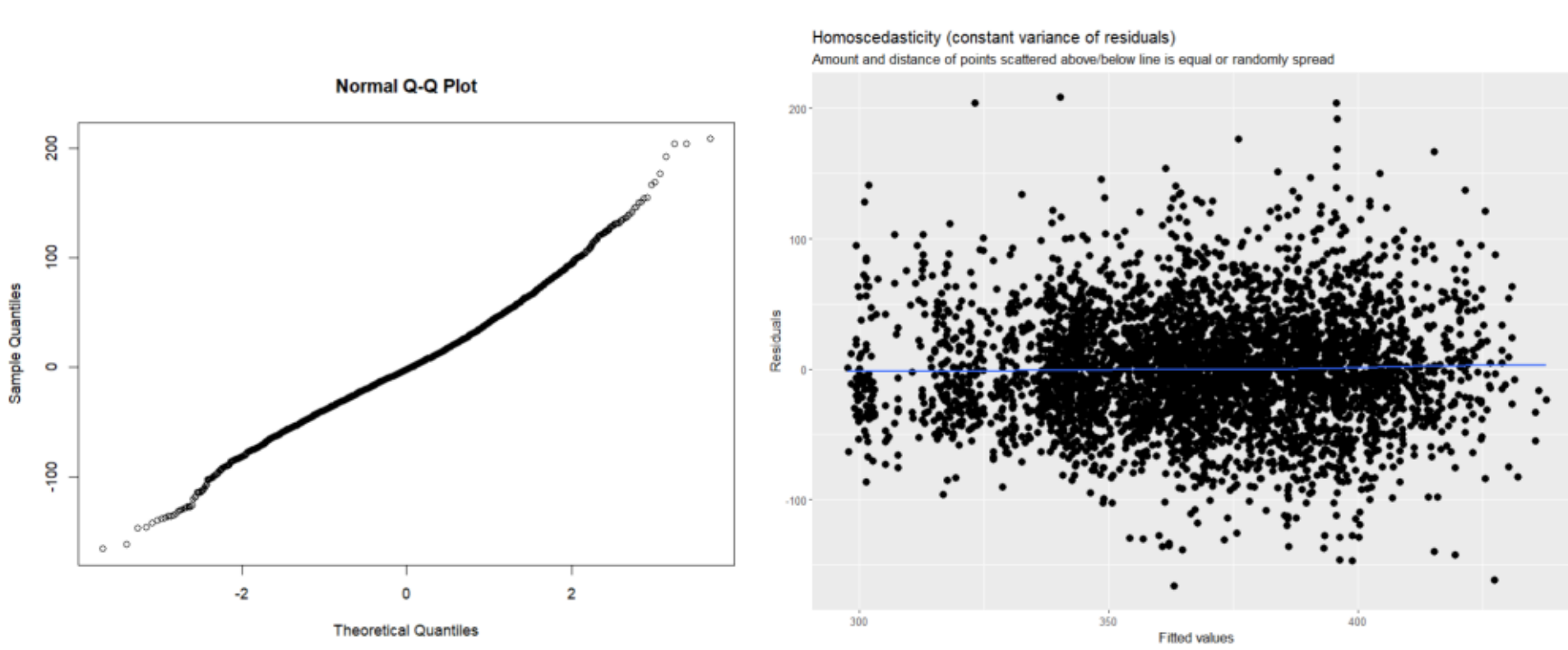

Note. Visual inspection of the residuals gave no reason for concern.

Table 10. Standardized beta coefficients

Parameter	Std. Coef.	95% CI
Intercept	0.55	[0.36, 0.74]
SSD sp. 1	-1.17	[-1.44, -0.91]
SSD sp. 2	-0.41	[-0.54, -0.28]
Stop outcome	0.21	[0.17, 0.24]
Sequence	-0.02	[-0.04, 0.01]

Note. Method: Refit

Table 11. Pseudo-R^2 coefficients

	Effect	Rsq	95% CI
1	Model	0.070	[0.057, 0.086]
2	Stop outcome	0.040	[0.030, 0.052]
3	SSD	0.035	[0.025, 0.046]
4	Sequence	0.000	[0.000, 0.002]

Note. Estimated marginal and conditional R^2: ~7% and ~31%.

3.3 Dependent variable: P3 amplitude

Figure 13

Probability density function of P3 amplitudes

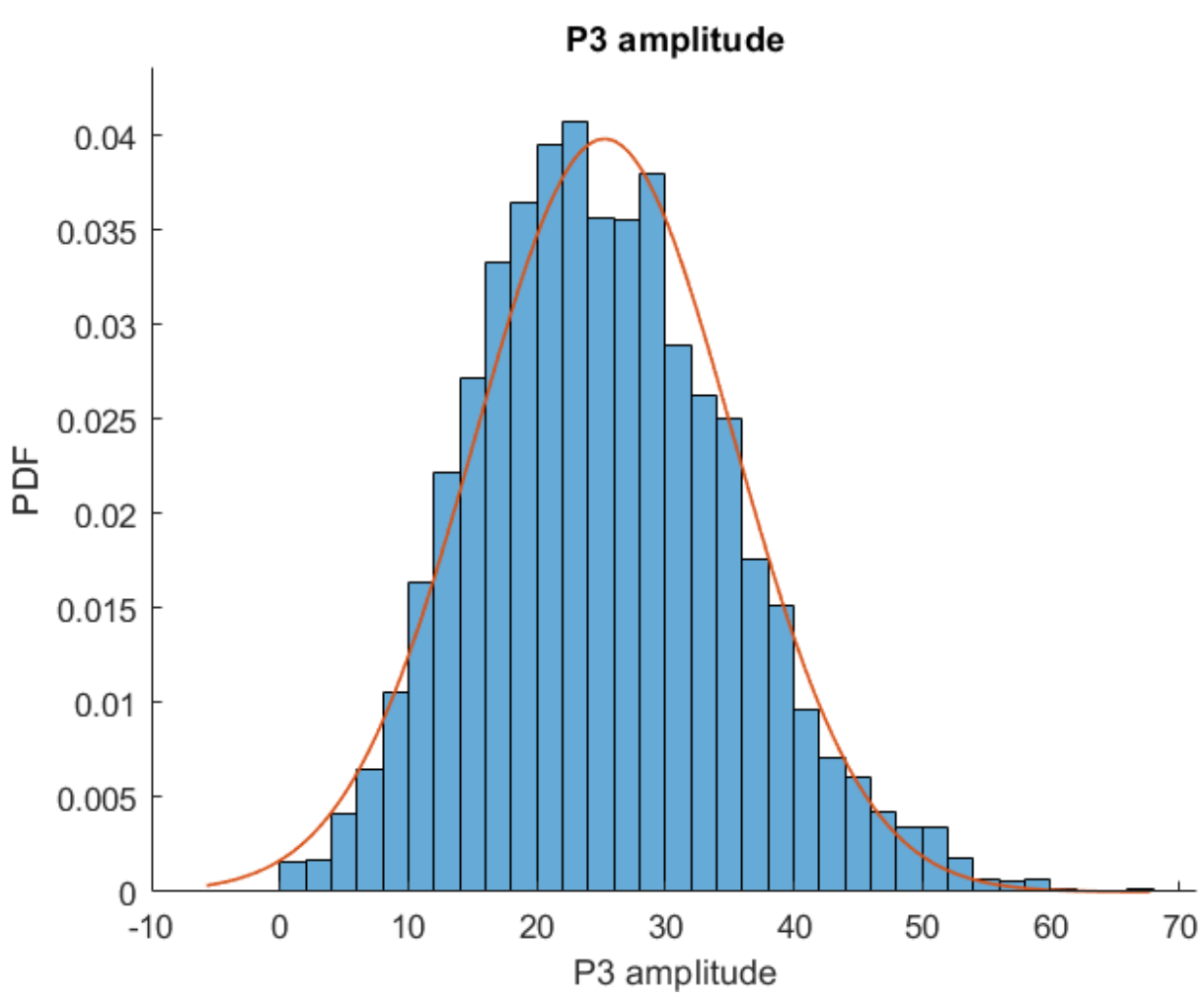

Note. Visual inspection of the raw scores and the fitted density function indicated a normal distribution.

Intra-class correlation coefficient:

The intra-class correlation coefficient of the null model was ~29%.

Table 12. Model comparisons

	Numpar	AIC	BIC	logLik	-2*log(L)	χ^2	*df*	*p*
Comparison of null Model vs. Model 1								
ModNull	3	31428	31447	-15711	31422			
Mod 1	8	31221	31272	-15602	31205	217.47	5	< 0.001
Comparison of Model 1 vs. Model 2								
Mod 1	8	31221	31272	-15602	31205			
Mod 2	13	31110	31193	-15542	31084	120.60	5	< 0.001
Comparison of Model 2 vs. Model 3								
Mod 2	13	31110	31193	-15542	31084			
Mod 3	17	31029	31137	-15497	30995	89.073	4	< 0.001
Comparison of Model 3 vs. Model 4								
Mod 4	10	31169	31233	-15575	31149			
Mod 3	17	31029	31137	-15497	30995	154.56	7	< 0.001
Comparison of Model 3 vs. Model 5								
Mod 5	10	31161	31225	-15571	31141			
Mod 3	17	31029	31137	-15497	30995	146.63	7	< 0.001
Comparison of Model 3 vs. Model 6								
Mod 6	10	31113	31177	-15547	31093			
Mod 3	17	31029	31137	-15497	30995	98.34	7	< 0.001
Comparison of Model 3 vs. Model 7								
Mod 7	13	31068	31151	-15521	31042			
Mod 3	17	31029	31137	-15497	30995	47.02	4	< 0.001

Results from comparing different random effect structures using likelihood ratio tests indicated that model 3 provided a better model fit. The selected random effect structure:

Model 3: 1 + SSD + Sequence + label | ID

Visualizing the model:

Visualizing the predicted slopes against the P3 amplitudes suggested a complex nonlinear relationship. Adding splines with increasing curvature would be too complex and a linear relationship could still capture the overall trend. The results from the P3 analysis should be interpreted with this caveat in mind.

A likelihood ratio test indicated that retaining the interaction between SSD and Stop outcome provided a better model fit, $\chi(1) = 6.28$, $p < .012$, whereas no statistical difference was found for the model without the interaction between Sequence and Stop outcome, $p = .13$. The model without the interaction between Sequence and Stop outcome was selected. We again tested different random effect structures and found that likelihood ratio tests indicated that retaining the random effects structure from above provided a better model fit.

The final model: ~ 1 + SSD * Stop outcome + Sequence + Stop outcome + (1+ SSD + Sequence + Stop outcome | ID)

Figure 14

The first subplot shows the QQ-norm of the residuals, while the second shows the fitted values against the residuals

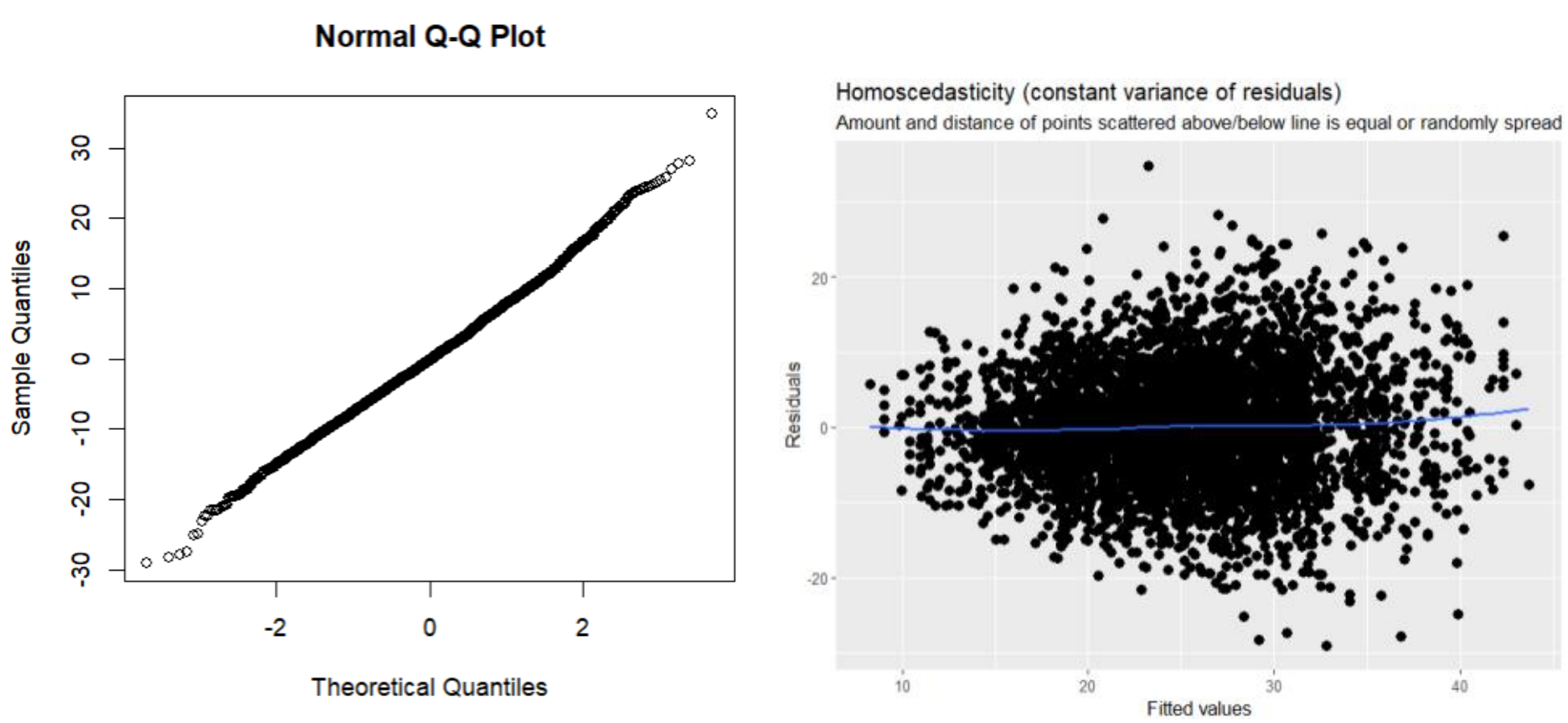

Note. Visual inspection of the residuals gave no reason for concern.

Table 13. Standardized beta coefficients

Parameter	Std. Coef.	95% CI
Intercept	3.80e-03	[-0.17, 0.18]
SSD	9.42e-03	[-0.06, 0.08]
Stop outcome	0.05	[0.00, 0.10]
Sequence	0.16	[0.11, 0.20]
SSD * Stop outcome	-0.04	[-0.08, -0.01]

Note. Method: Refit

Table 14. Pseudo-R^2 coefficients

	Effect	Rsq	95% CI
1	Model	0.031	[0.022, 0.042]
2	Sequence	0.023	[0.015, 0.032]
3	Stop outcome	0.003	[0.000, 0.007]
4	SSD * Stop outcome	0.002	[0.000, 0.005]
5	SSD	0.000	[0.000, 0.002]

Note. Estimated marginal and conditional R^2: ~3% and ~41%.

Follow-up testing of significant interaction between SSD and Stop outcome using Tukey corrected simple slope analysis.

Table 15. Mean differences with Tukey corrected p-values

Level	Estimate	SE	df	t-ratio	p
US	-0.130	0.159	79.2	-0.816	0.417
SS	0.202	0.144	52.9	1.408	0.165
US - SS	-0.332	0.132	376	-2.509	0.013

Note. Satterthwaite degrees of freedom. Confidence interval = 0.95.

4 Simulation analysis for peak extraction procedure

All simulations were based off the scripts used to simulate data in Huster and colleagues (2015). We simulated 100 trials of ERP signals. For each of these trials, scaled pink noise were added (scaled by a factor of 4) using the Matlab function “pinknoise”. Pink noise was bandpass filtered between 2 and 20Hz. We varied temporal onset times in windows of 40ms around the zero-point yielding a window size of 80ms using a uniform distribution. Amplitudes were scaled between randomly drawn integers from 0.4 to 1.5.

Figure 15

1. *Before latency minimization*

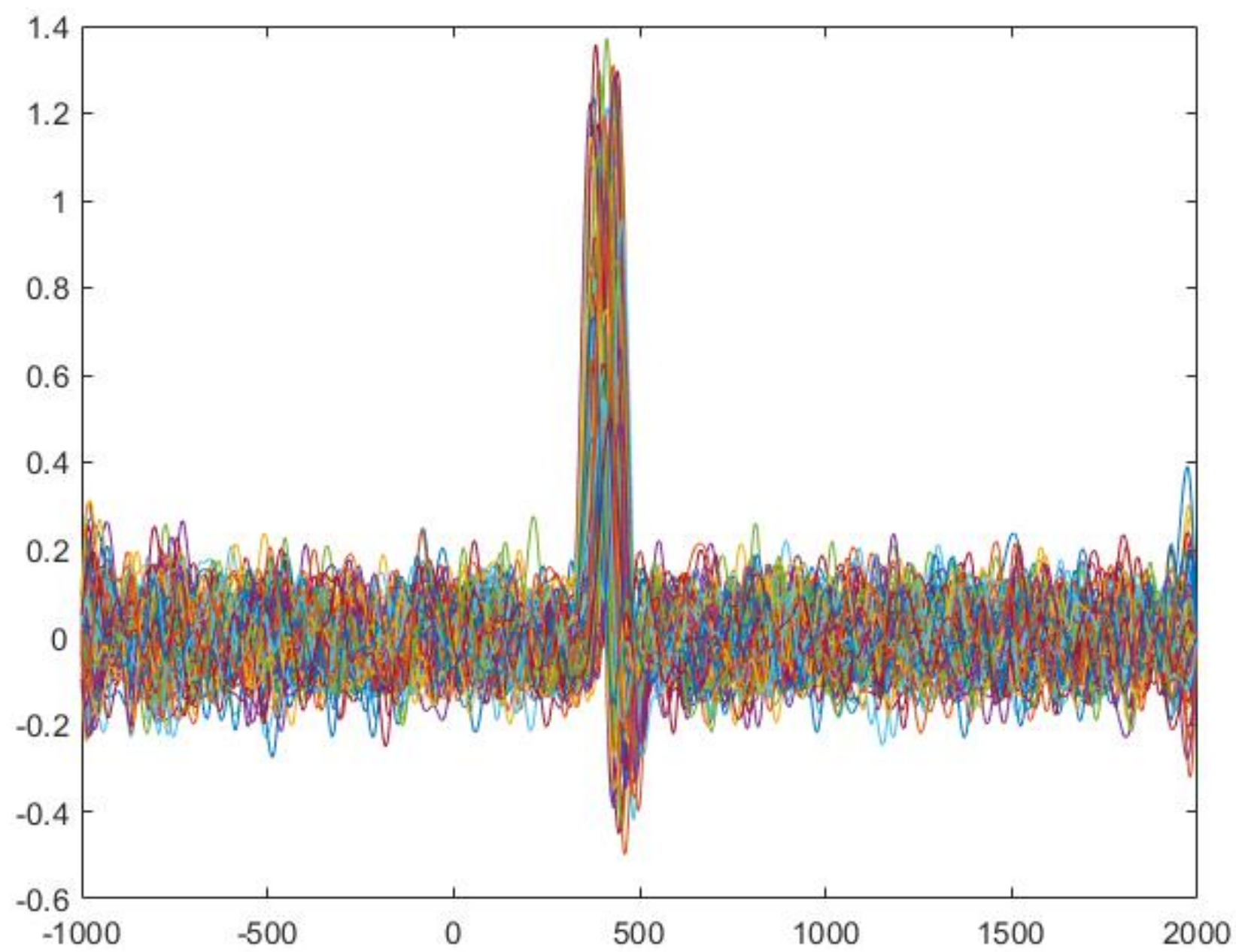

Note. The simulated single trials vary in both amplitude and latency relative to the zero point.

2. *After latency minimization*

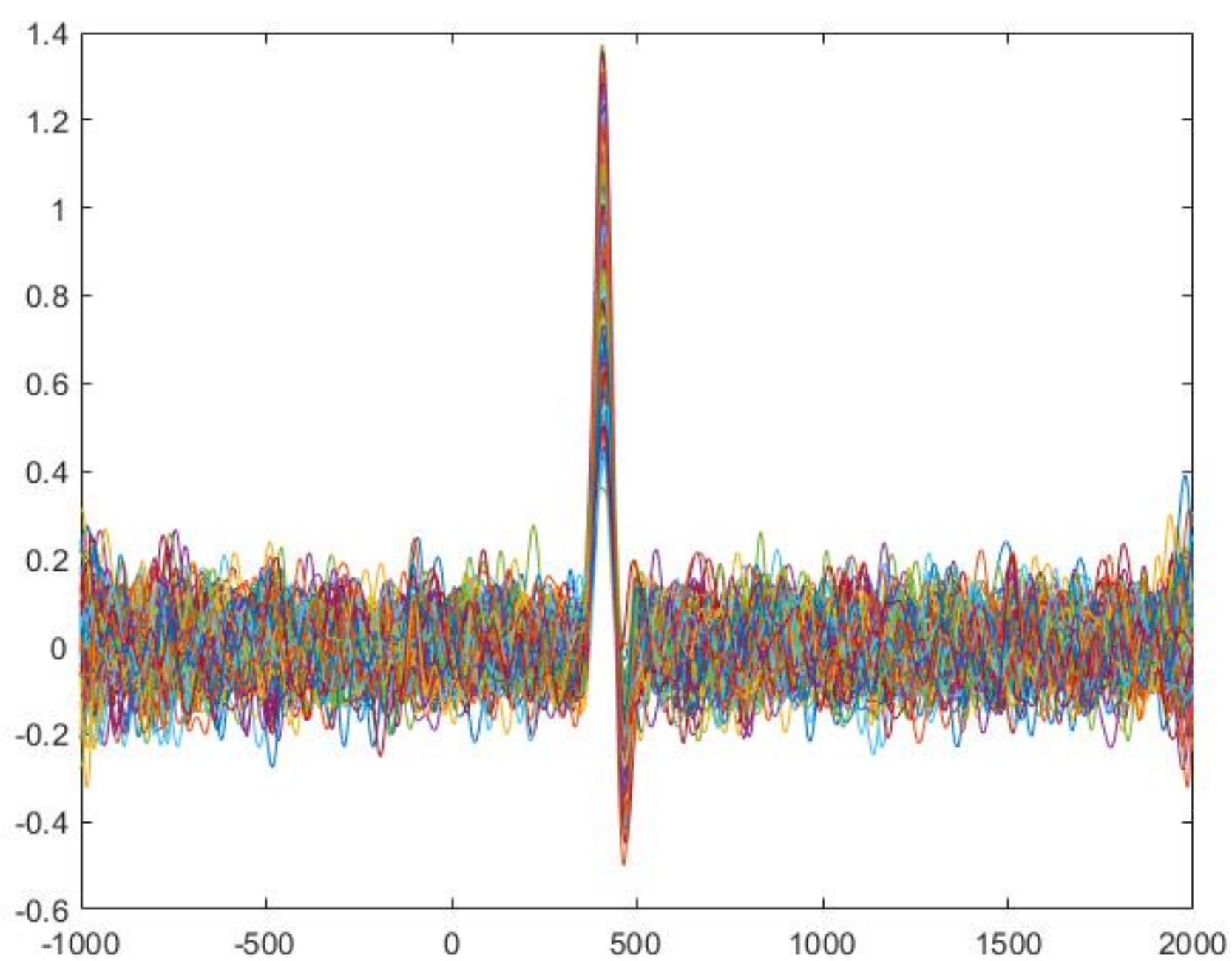

Note. After latency minimization, trials are shifted such that they align temporarily decreasing the window size for subsequent peak detection.

Figure 15

ERPs calculated before and after latency minimization

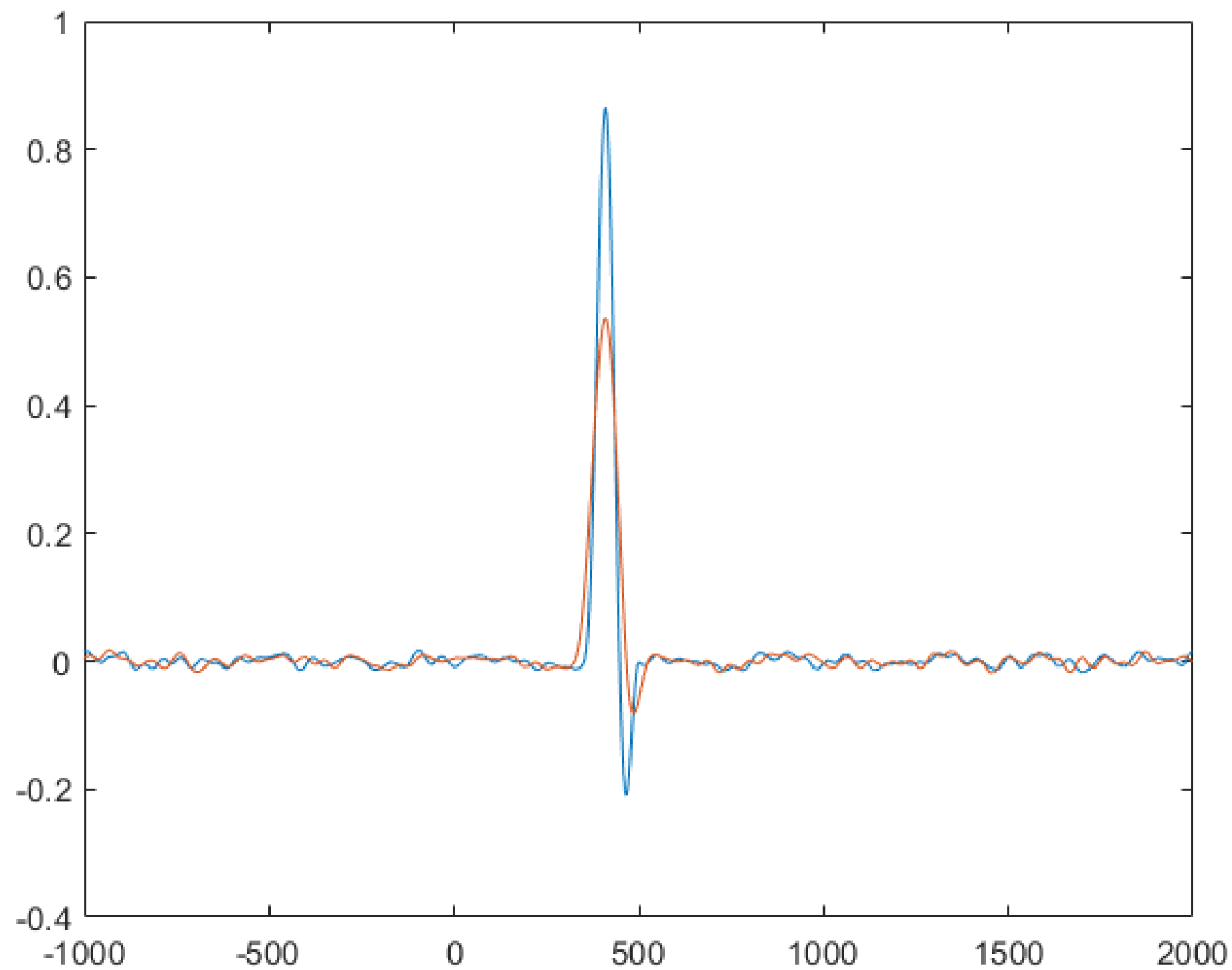

Note. The red line indicates the ERP before latency minimization, the blue line after latency minimization.

Figure 16

Simulated signal, one trial

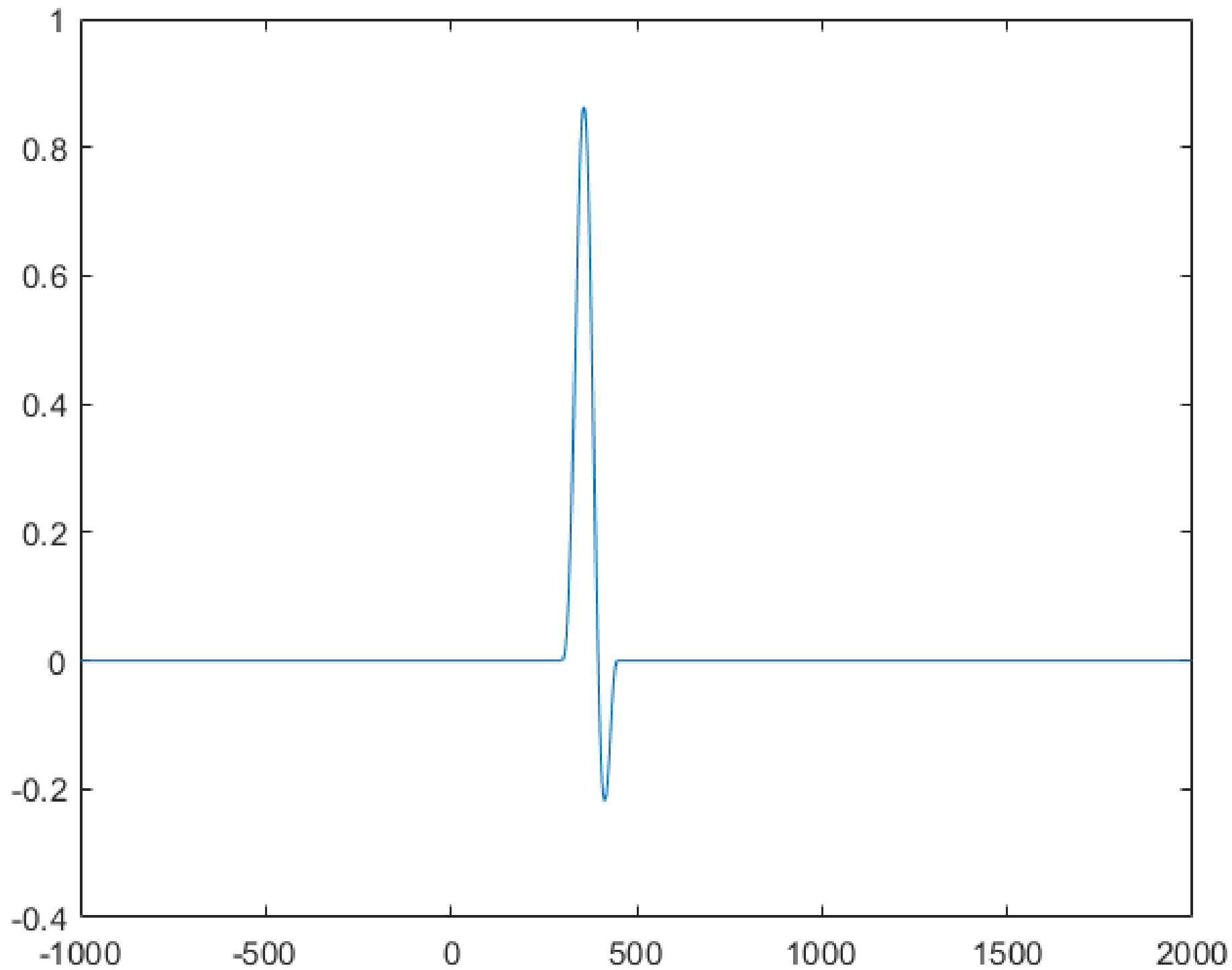

Note. The ERP before latency minimization is a smeared-out version of the simulated shape due to latency jitter.

Peak detection. Across many studies, the searcher window is determined based on the ERP and predetermined upper and lower limits. It is common to hold the upper and lower limits identical across participants. If any trial should fall outside of this window, then the peak detection procedure would not be able to estimate the peak. By minimizing latency variation in single trials, the window to search for peaks is made smaller. Because trials are now temporally aligned, the peaks should be tightly clustered around the ERP peaks. Our procedure identifies the probable peak from the latency minimized data at single trial level. Because we know how much each single trial has been shifted, we can easily calculate where in the unshifted data the identified peak occurred. It makes it easier to identify peaks given a narrower searcher window. Our procedure would not be necessary in the case where most/all single trials occur within a narrow time range following stimulus onset.

Experiences. Simulated temporal variation follows a uniform distribution. This should decrease the performance of the procedure given that the procedure relies on clustering around a central tendency. In real data, many trials will occur around the same timepoint

with roughly similar shapes (i.e., central tendency). Once the temporal variation is large enough, the initial template (being the ERP) will be a smeared version often visually looking like two ERPs immediately following each other (see figure below).

Figure 17

Large temporal variation results in a smeared ERP

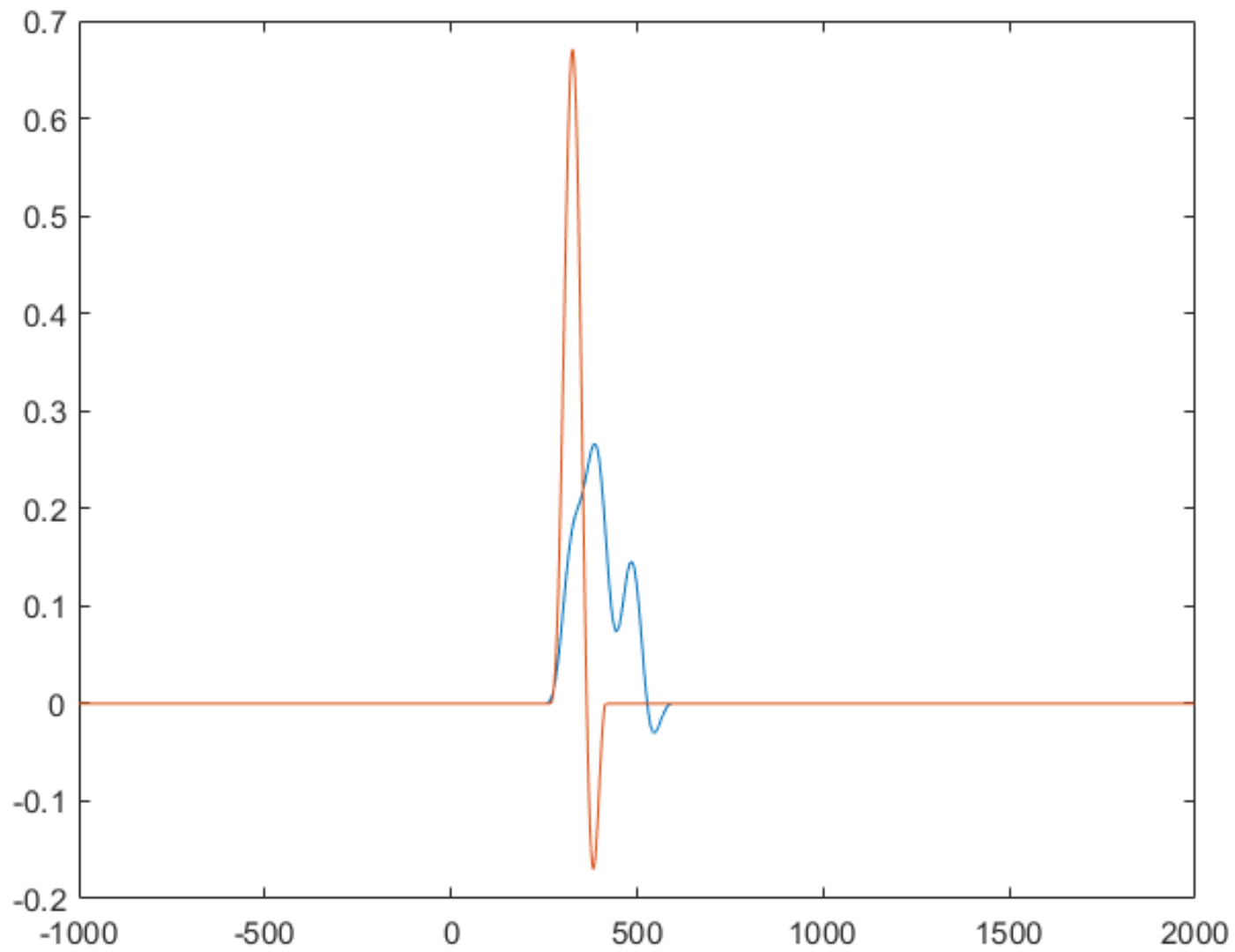

Note. The orange signal is one single trial. The blue signal is the average across all single trials.

5 Electrode layout 32 channels acticap

Figure 18

Electrode layout

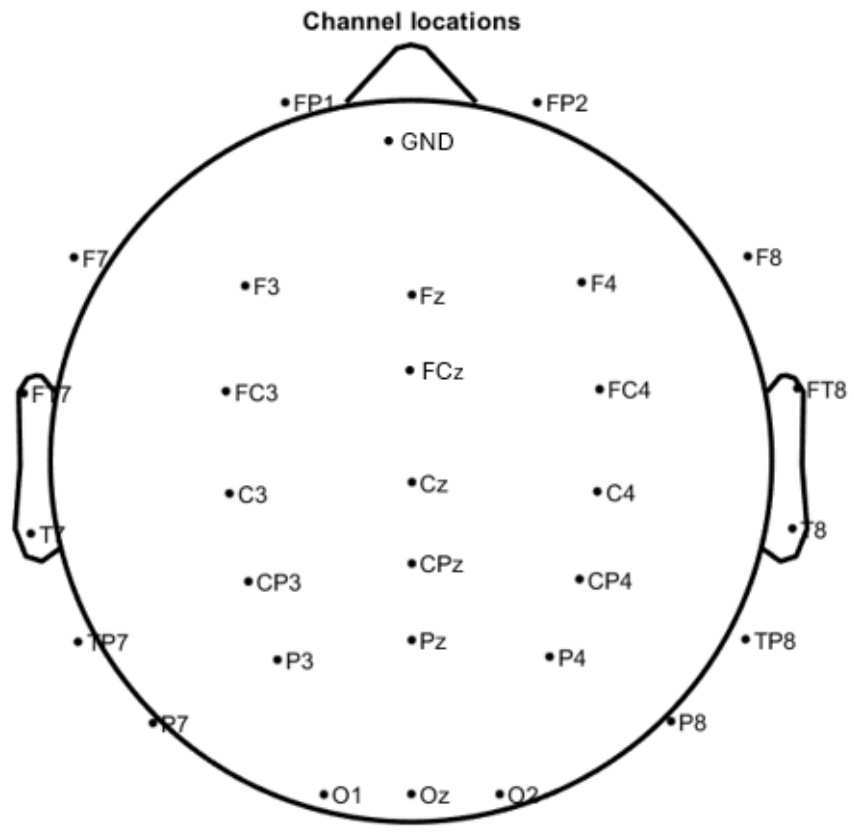

6 Psychtoolbox VBLSyncTest results

60Hz – 16.67ms PTB missed 1 out of 600 stimulus presentation deadlines. Average time delta between return from swap completion and return of flip in milliseconds is about 0.021ms.

7 Sensitivity analysis

We fitted alternative model specifications for each of the final models to evaluate whether the main conclusions changed. For each final model, we compared a simpler random effect structure by fitting a random-intercept-only model and evaluated the significance of each predictor variable using type III ANOVAs.

N2 latency

Table 1. Type III ANOVA sensitivity analysis results (Satterthwaite) for N2 latency

Effect	F	df num	df den	*p*
SSD	63.83	2	3601	<.001 ***
Stop outcome	33.61	1	4357	<.001 ***
Sequence	0.21	1	4331	.65
SSD*Stop outcome	3.84	2	4354.8	.02 *

Significant codes: *** 0.001, ** 0.01, * 0.05.

Summary N2 latency sensitivity analysis. The estimated fixed effects from the by-random-intercept-only model remained largely similar to the final model. Estimated

significance shrunk for the interaction term. However, the main conclusions remained the same. Residuals and homoscedasticity were visually inspected and gave no reason for concern. A likelihood ratio test indicated that the final model provided a better fit compared with the by-intercept-only model, $\chi(5) = 20.46$, $p < 0.001$.

N2 amplitude

Table 2. Type III ANOVA sensitivity analysis results (Satterthwaite) for N2 amplitude

Effect	F	df num	df den	*p*
SSD	72.28	2	4241.8	<.001 ***
Stop outcome	250.84	1	4355.4	<.001 ***
Sequence	39.48	1	4328.4	<.001 ***
Sequence*Stop outcome	7.89	1	4329.1	.005 **

Significant codes: *** 0.001, ** 0.01, * 0.05.

Summary N2 amplitude sensitivity analysis. The estimated fixed effects from the by-random-intercept-only model remained largely similar to the final model. All signs remained similar and coefficients remained significant. Residuals and homoscedasticity were visually inspected and suggested that the resulting model did deviate from assumptions underlying the analysis, however, not to a large extent. A likelihood ratio test indicated that the final model provided a better fit compared with the by-intercept-only model, $\chi(5) = 153.84$, $p < 0.001$.

P3 latency

Table 3. Type III ANOVA sensitivity analysis results (Satterthwaite) for P3 latency.

Effect	F	df num	df den	*p*
SSD	44.33	2	4130.7	<.001 ***
Stop outcome	227.51	1	4392.4	<.001 ***
Sequence	2.18	1	4360.1	.141

Significant codes: *** 0.001, ** 0.01, * 0.05.

Summary P3 latency sensitivity analysis. The estimated fixed effects from the by-random-intercept-only model remained highly similar to the coefficients from the final model. All signs remained similar and coefficients remained significant. Residuals and homoscedasticity were visually inspected and gave no reason for concern. A likelihood ratio test indicated that the final model provided a better fit compared with the by-intercept-only model, $\chi(2) = 17.32$, $p < 0.001$.

P3 amplitude

Table 4. Type III ANOVA sensitivity analysis results (Satterthwaite) for P3 amplitude.

Effect	F	df num	df den	*p*
SSD	1.15	1	4058.8	.284
Stop outcome	17.84	1	4387.8	<.001 ***
Sequence	152.37	1	4359.3	<.001 ***
SSD * Stop outcome	16.6	1	4369.1	<.001 ***

Significant codes: *** 0.001, ** 0.01, * 0.05.

Summary P3 amplitude sensitivity analysis. The estimated fixed effects from the by-random-intercept-only model remained highly similar to the coefficients from the final model. Reducing the model to include only a random intercept resulted in larger change in significance level, especially for the predictor variable stop outcome. A follow-up analysis of the significant interaction indicated that a significant linear increase in P3 amplitude with increasing SSD, $p = 0.001$. The slope for unsuccessful stopping remained non-significant, $p = 0.227$. Residuals and homoscedasticity were visually inspected and gave no reason for concern. Again, like with the full model, the association with SSD should be interpreted with caution as the relationship was highly nonlinear. The estimated coefficient for stop outcome was significant in the random-intercept-only model, whereas the value of the coefficient remained largely the same. The estimated standard error for the predictor variable in the reduced model is roughly half the standard errors in the full model. A likelihood ratio test indicated that the final model provided a better fit compared with the by-intercept-only model, $\chi(9) = 211.53$, $p < 0.001$. This adds complexity to the interpretation of the sensitivity analysis. Together, the results indicate that the full model

was likely too complex, potentially leading to inflated standard errors and failing to show the real effect by overfitting random effects. It is possible that the estimates from the full model were overly conservative and that more observations (i.e., trials) are needed to be more confident in the estimated effects.

References

Huster, R. J., Plis, S. M., & Calhoun, V. D. (2015). Group-level component analyses of EEG: validation and evaluation. *Frontiers in neuroscience*, *9*, 254.
